# BAP1 suppresses prostate cancer progression by deubiquitinating and stabilizing PTEN

**DOI:** 10.1002/1878-0261.12844

**Published:** 2020-11-20

**Authors:** Rong Deng, Yanmin Guo, Lian Li, Jianfeng He, Zhe Qiang, Hailong Zhang, Ran Chen, Yanli Wang, Xian Zhao, Jianxiu Yu

**Affiliations:** ^1^ Department of Biochemistry and Molecular Cell Biology State Key Laboratory of Oncogenes and Related Genes Shanghai Key Laboratory of Tumor Microenvironment and Inflammation Shanghai Jiao Tong University School of Medicine Shanghai China; ^2^ Basic Clinical Research Center Renji Hospital School of Medicine Shanghai Jiao Tong University Shanghai China

**Keywords:** BAP1, cancer progression, deubiquitination, prostate cancer, PTEN

## Abstract

Deubiquitinase BAP1 is an important tumor suppressor in several malignancies, but its functions and critical substrates in prostate cancer (PCa) remain unclear. Here, we report that the mRNA and protein expression levels of BAP1 are downregulated in clinical PCa specimens. BAP1 can physically bind to and deubiquitinate PTEN, which inhibits the ubiquitination‐mediated degradation of PTEN and thus stabilizes PTEN protein. Ectopically expressed BAP1 in PCa cells increases PTEN protein level and subsequently inhibits the AKT signaling pathway, thus suppressing PCa progression. Conversely, knockdown of BAP1 in PCa cells leads to the decrease in PTEN protein level and the activation of the Akt signaling pathway, therefore promoting malignant transformation and cancer metastasis. However, these can be reversed by the re‐expression of PTEN. More importantly, we found that BAP1 protein level positively correlates with PTEN in a substantial fraction of human cancers. These findings demonstrate that BAP1 is an important deubiquitinase of PTEN for its stability and the BAP1‐PTEN signaling axis plays a crucial role in tumor suppression.

## Graphical abstract

PTEN protein levels are regulated through ubiquitination. Here, we identify BAP1 as a deubiquitinase that physically binds to and deubiquitinates PTEN, thereby inhibiting PTEN degradation. Stabilized PTEN suppresses prostate cancer progression by inhibiting Akt phosphorylation.

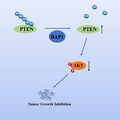

**Abbreviations**
BAP1: the BRCA1‐associated protein 1
DUBs: deubiquitinases
GEO: Gene Expression Omnibus
IP: immunoprecipitation
KEGG: the Kyoto Encyclopedia of Genes and Genomes
PCa: prostate cancer
PTEN: phosphatase and tensin homolog deleted on chromosome 10
qRT–PCR: quantitative real‐time polymerase chain reaction
TCGA: the Cancer Genome Atlas
USP: the ubiquitin–proteasome system
VM: vasculogenic mimicry

## 1 Introduction

Prostate cancer (PCa) is the most commonly diagnosed cancer and the third leading cause of cancer‐related death in men in the United States [1, 2]. The causes of PCa have been extensively studied but are still poorly understood. Some known alterations of cancer‐related genes/proteins have been described as key drivers and potential biomarkers in PCa progression, and a series of screening and diagnosis markers have recently emerged; however, the sensitivities and specificities of those in detecting high‐grade PCa were limited [3]. Thus, the mechanisms underlying tumorigenesis and cancer progression still need to be deeply explored so as to provide better strategies for diagnosis and treatment.

The ubiquitin–proteasome system (UPS) plays important roles in diverse physiological and pathological processes by mostly controlling the turnover of substrate proteins [4]. Ubiquitination is a dynamic and reversible process, which is deconjugated by a large family of deubiquitinases (DUBs) [5, 6]. DUBs affect the degradation, activation, and localization of target proteins through specifically removing ubiquitin chains, which is implicated in various signaling pathways and cell homeostasis [7, 8, 9]. Either dysfunction or dysregulation of DUBs is closely related to tumorigenesis and the development of multiple cancers [10, 11], and targeting DUBs is a good anticancer therapeutic strategy [12, 13]. BRCA1‐associated protein 1 (BAP1) is a member of the ubiquitin C‐terminal hydrolases (UCH) subfamily of DUBs, which is initially characterized as a deubiquitinase regulating the function of BRCA1 [14]. BAP1 consists of an N‐terminal UCH (ubiquitin carboxyl hydrolase) domain, a HCF (host cell factor)‐binding domain HBM in the middle and two NLS (nuclear localization signal) motifs at the C terminus [15]. BAP1 acts as a deubiquitinase for various proteins including H2A [16, 17, 18], HCF‐1 [19], INO80 [20], KLF5 [21], MCRS1 [22], IP3R3 [23], gamma‐tubulin [24], OGT [25], and BAP1 itself [26], which are involved in diverse cellular processes including chromosome stability, cell proliferation, cell cycle, and apoptosis. Further, BAP1 interacts with several transcription factors such as FOXK1/2 [27] and YY1 [28] to regulate gene expression. BAP1 is emerging as an important tumor suppressor in human cancers [29, 30], which requires its deubiquitinating activity and nuclear localization [31]. Growing evidences show that BAP1 is frequently mutated in many human cancers to suggest BAP1 as a tumor suppressor [32, 33, 34, 35, 36, 37, 38, 39, 40]; however, there are also a few studies reporting that BAP1 plays a role in promoting growth of melanoma [41] and breast cancer [21]. So far, functions and regulations of BAP1 in PCa are largely unexplored.

In this study, we demonstrated that BAP1 acted as a tumor suppressor in PCa progression. More importantly, we characterized its critical role in tumor suppression by deubiquitinating and stabilizing PTEN that is a well‐established tumor suppressor in PCa. Furthermore, relevance analyses of clinical data in PCa and many other cancers revealed a significant positive correlation between BAP1 and PTEN at the protein level, indicating that the BAP1‐PTEN signaling axis might play important roles in tumor suppression.

## 2 Materials and methods

### 2.1 Antibodies

Mouse anti‐Akt1 (#2967), anti‐ubiquitin (#3936), anti‐Myc (#2276), rabbit anti‐BAP1 (#13271), anti‐PTEN (#9559), and phospho‐Akt‐S^473^ (#4060) antibodies were purchased from Cell Signaling Technology (Danvers, MA, USA). Rabbit anti‐BAP1 (#10398‐1‐AP) and mouse anti‐beta‐actin (#60008‐1‐Ig) antibodies were purchased from Protein Technology (Rosemont, IL, USA). Mouse anti‐PTEN (ab170941) antibody was purchased from Abcam (Cambridge, UK). Mouse anti‐beta Tubulin (100109‐MM05T) antibody was purchased from Sino Biological (Beijing, China). Anti‐Flag M2 (F1804) antibody was purchased from Sigma‐Aldrich (St. Louis, MO, USA). Anti‐HA (16B12) antibody was purchased from Covance (Berkeley, CA, USA). Anti‐GST (#M0807‐1) antibody was purchased from HuaBio (Hangzhou, Zhejiang, China). Protein G Plus/A agarose suspension (#IP05) was purchased from Calbiochem (San Diego, CA, USA).

### 2.2 Plasmids

Plasmids Flag‐PTEN, pCDH513B‐PTEN, and GST‐PTEN were described previously [42]. Full‐length BAP1 was inserted into pEF‐HA, pCDH513B, pGEX4T‐1, and pET‐28a vectors. The mutant BAP1^C91S^ was generated by PCR‐directed mutagenesis with KOD‐Plus‐Mutagenesis Kit (TOYOBO, Tokyo, Japan). The shRNAs targeting *BAP1* 3′‐UTR and *AKT1* were cloned into the vector pLKO.1. The truncated PTEN and BAP1 plasmids were obtained by mutagenesis PCR or subclone. All plasmids were checked by sequencing. Primer sequences for plasmids construction, shRNAs, and siRNAs were listed in Table S1.

### 2.3 Cell culture, transfection, and stable cell line establishment

HEK293T, 293FT, and HeLa cells were cultured in DMEM containing 10% fetal bovine serum (FBS) supplemented with penicillin and streptomycin at 37 °C and 5% CO_2_. DU145 and PC3 cells were cultured in RPMI‐1640 medium supplemented with 10% FBS. P69 and M12 were cultured in RPMI‐1640 medium containing 5% FBS, supplemented with 10 ng·mL^−1^ epidermal growth factor (EGF), 0.2 μm dexamethasone, 5 μg·mL^−1^ insulin, 5 μg·mL^−1^ transferrin, 5 μg·mL^−1^ sodium selenite, 50 μg·mL^−1^ gentamicin, and 100 U·mL^−1^ penicillin/streptomycin. Plasmid transfection into 293T and 293FT cells was performed using polyethylenimine (PEI) according to manufacturer’s instructions. To establish stable cell lines, the lentiviral vector carrying BAP1, PTEN, or shRNA sequence together with the packaging plasmids (pMD2G and pCMVdR8) was transfected into 293FT cells using PEI. The supernatants were harvested 48 h later and centrifuged at 2500 ***g*** for 10 min. DU145, P69, and M12 cells were incubated with viral supernatants in the presence of 5 µg·mL^−1^ polybrene for 24 h. Stable cell lines were selected with 5–10 μg·mL^−1^ puromycin for 3–4 days, and the expression levels were analyzed by western blotting.

### 2.4 Immunoprecipitation (IP) and GST pull‐down assays

293T or HeLa cells transfected with indicated plasmids were lysed in lysis buffer (50 mm Tris/HCl pH7.4, 150 mm NaCl, 0.5% NP‐40, 2 mm EDTA, 0.05% SDS, 0.5 mm DTT, and complete protease inhibitor cocktail) on ice for 1 h. 1 mg of lysates was incubated with protein A/G‐agarose beads and specific antibodies overnight at 4 °C. The complexes bound to agarose beads were washed 5 times in the same lysis buffer and subjected to 8% SDS/polyacrylamide gels for western blotting analysis.

For immunoprecipitation under denaturing conditions, cells were lysed in SDS‐lysis buffer (50 mm Tris/HCl pH7.4, 150 mm NaCl, 1% SDS, and 5 mm DTT) and then boiled for 10 min. The lysates were clarified by centrifugation at 16 000 ***g*** for 10 min at 4 °C. The clarified samples were diluted into 0.1% SDS and 0.5 mm DTT with dilution buffer (50 mm Tris/HCl pH7.4, 150 mm NaCl, 0.5% Triton X‐100, 2 mm EDTA, and complete protease inhibitor cocktail). The soluble supernatant fractions were harvested and subjected to immunoprecipitation experiments as described above.

For GST pull‐down assay, purified GST or GST‐PTEN was incubated with GST beads and indicated cell lysates or recombinant proteins at 4 °C. GST beads were then washed three times with lysis buffer. The bound proteins were analyzed by western blotting.

### 2.5 Immunofluorescence (IF) staining

HeLa cells seeded on coverslips were transfected with the indicated plasmids using Lipofectamine 2000. At 24 h after transfection, cells were fixed with 4% paraformaldehyde. Following incubation in blocking solution, cells were stained with the anti‐HA antibody and then incubated in the second antibody (Alexa Fluor^®^ 568). DU145 stable cells with PTEN overexpression were fixed with 4% paraformaldehyde and then stained with primary antibodies (anti‐PTEN and anti‐BAP1) and subsequently with secondary antibodies (Alexa Fluor^®^ 488 or Alexa Fluor^®^ 568). Nuclei were stained with DAPI. Images were acquired using Leica TSC SP8 confocal microscope.

### 2.6 **qRT**–**PCR**

qRT–PCR was performed according to our previous protocol [43]. Total RNAs were extracted using TRIzol reagent (Invitrogen, Carlsbad, CA, USA) following the instructions. 1 μg of RNAs was used for reverse transcription by using PrimeScript™ RT–PCR Kit (Takara, Otsu, Shiga, Japan). *PTEN* mRNA levels were analyzed by using the SYBR‐Green Master PCR Mix (Applied Biosystems) with an ABI StepOne system (Applied Biosystems, Foster City, CA, USA). GAPDH was used for normalization of *PTEN* mRNA. The primers for qRT–PCR were listed in Table S1.

### 2.7 Vasculogenic mimicry (VM) formation and three‐dimensional (3D) culture growth assays

The vasculogenic mimicry formation was performed as described before [44]. Briefly, prethawed Matrigel matrix™ (#3445‐005‐01, Trevigen, Gaithersburg, MD, USA) was added into the inner well of µ‐slides (Ibidi Gmbh, Martinsried, Germany) and incubated at 37 °C for 1 h. 50 μL of cells (1 × 10^5^ cells·mL^−1^) was seeded onto the polymerized matrix. 3D culture growth assay was conducted as described in our previous study [45]. 5 μL of cell solutions (1 × 10^5^ cells·mL^−1^) mixed with 5 μL of matrix gel was transferred into a well of µ‐slides and incubated at 37 °C for 1 h. The complete cell culture medium was added onto the solidified gel, and the µ‐slides were incubated at 37 °C. Microscopy images were taken after three days.

### 2.8 Migration assay by wound healing

We used a previously described method to analyze cell migration [46]. Briefly, DU145, P69, or M12 stable cells were seeded into the well of 12‐well plates and cultured until 90% confluence. Scratches were made with sterile pipette tips. The cells were cultured in complete medium. Photographs were captured at the indicated times.

### 2.9 Migration assay with RTCA‐DP

The protocol for the migration assay with the xCELLigence RTCA‐DP system (Roche) was described previously [45]. Briefly, cells were serum starved for 4–6 h, then detached with trypsin, and resuspended in serum‐free medium with concentration of 1 × 10^5^ cells·mL^−1^. 100 μL of suspension was seeded into the pre‐equilibrated upper chamber of the CIM plate, and the low chamber was added complete medium for migration. Cell index values were detected every 15 min following procedure. RTCA software v1.2 (Roche Applied Science, Indianapolis, IN, USA) was used to calculate the slopes of the curves at various time points.

### 2.10 Soft‐agar colony formation assay

The soft‐agar colony formation assay was performed as described before [42]. Briefly, the agar‐medium mixture containing 0.6% low‐melting point agarose (Amresco) and 5% FBS was added in six‐well plate as the base agar gel, and cells resuspended in 0.35% agar (Amresco, Wayne, PA, USA) with 5% FBS were seeded onto top of the base gel as the colony formation gel, and then incubated at 37 °C. After 3–4 weeks, cell colonies were fixed with methanol and stained with 0.005% Crystal Violet. The photographs of the colonies were taken, and the number of colonies was scored.

### 2.11 Xenograft tumor model

The experiment of xenograft tumor model was conducted as described before [47]. Each of stable DU145 cell lines (at the concentration of 2.5 × 10^7^ cells·mL^−1^) was injected subcutaneously into 5‐week‐old male BALB/c nude mice (*n* = 6) on the bilateral backs. The tumor volume was measured at the indicated time. Mice were sacrificed after 28 days. Tumors were dissected, weighted, and photographed. All animal studies were conducted with the approval and guidance of Shanghai Jiao Tong University Medical Animal Ethics Committees.

### 2.12 Analyses of TCGA and GEO data

The online database Gene Expression Profiling Interactive Analysis (GEPIA, http://gepia.cancerpku.cn/index.html) was used to analyze the RNA sequencing expression of BAP1 in prostate adenocarcinoma (PRAD) patients based on The Cancer Genome Atlas (TCGA) and the Genotype‐Tissue Expression (GTEx) projects.

The reverse‐phase protein array (RPPA) data of BAP1 protein, PTEN protein, and p‐AKT(T308/S473) in TCGA tumor tissue sample were obtained from TCPA (The Cancer Proteome Atlas) which contains cancer proteomic datasets of 8167 tumor samples including all TCGA tumor tissue sample sets. The microarray gene expression profile datasets (accession ID: GSE23035) from Gene Expression Omnibus (GEO, https://www.ncbi.nlm.nih.gov/geo/) database were used to analyze in this study. The expression levels were normalized with Limma package by using r software. To identify differentially expressed genes, the fold change cutoff was set to 2 and the adjusted *P*‐value cutoff was selected as 0.05. The Kyoto Encyclopedia of Genes and Genomes (KEGG) pathway enrichment analysis was performed by the Database for Annotation, Visualization and Integrated Discovery (DAVID, https://david.ncifcrf.gov/home.jsp).

### 2.13 Statistical analysis

Statistical analyses were performed with graphpad prism version 7 (GraphPad Software, La Jolla, CA, USA) software package. Mouse xenografts and soft‐agar colony formation are presented as means ± standard error of the mean (SEM). Student’s *t*‐test (two‐tailed) was used to compare statistical significance between two groups. The correlation between BAP1 protein, PTEN protein, and p‐AKT(T308/S473) levels was analyzed by Pearson correlation test and linear regression. A value *P* < 0.05 was considered statistically significant. Most of experiments such as IP/WB, GST pull‐down assays, IF, VM, 3D culture, and soft‐agar colony formation were repeated at least 3 times.

## 3 Results

### 3.1 BAP1 is downregulated in human PCa

**Fig. 1:**
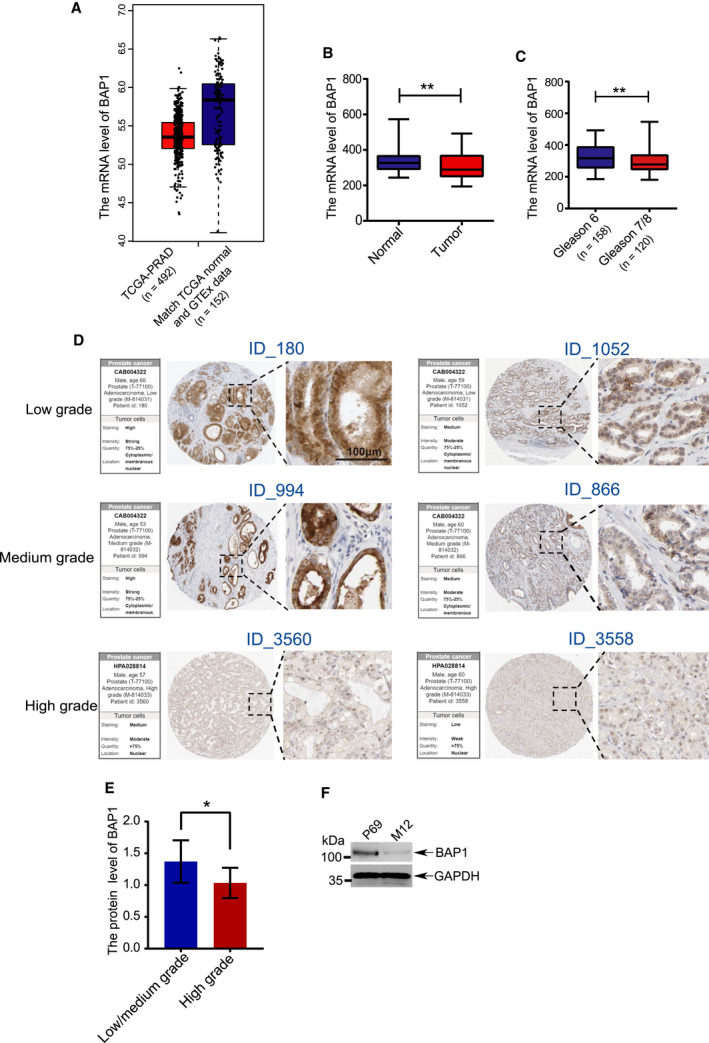
Dysregulation of BAP1 in PCa and cancer cell lines. (A) The mRNA levels of *BAP1* were compared between normal tissue specimens (*n* = 152) and PRAD specimens (*n* = 492), which derived from The Genotype‐Tissue Expression (GTEx) normal prostate tissue datasets and The Cancer Genome Atlas (TCGA) PRAD datasets. (B)The mRNA levels of BAP1 were compared between prostate normal and tumor tissues derived from R2 database, including normal tissues (*n* = 70) and tumor tissues (*n* = 262). (C) The mRNA levels of *BAP1* were compared based on PCa gleason scores. Gleason score 6 (*n* = 158), gleason score 6 and 7 (*n* = 120). (D) The representative images of IHC (immunohistochemistry) immunoblotted with BAP1 in PCa tissues collected from HPA (human protein atlas) database, including low, medium, and high‐grade PCa tissues. In each grade group, the representative images with strong or weak staining were presented, respectively. The high magnification insets of the images and the detailed information of staining were shown. Scale bars: 100 μm. (E) Quantification of the intensity of IHC by using the software ImageJ. *n* = 16 (low/medium grade), *n* = 26 (high grade). (F) The BAP1 protein levels were compared between in low‐tumorigenic P69 and highly tumorigenic M12 cell lines by using western blotting analysis. Error bars in B, C, and E indicated mean ± SD. Data analysis in B, C, and E was conducted by unpaired *t*‐test (*P < 0.05, **P < 0.01, ***P < 0.001).

To investigate the expression of *BAP1* in prostate cancer, the data were obtained from TCGA (The Cancer Genome Atlas) for analysis. The mRNA levels of *BAP1* were downregulated in the prostate adenocarcinoma (PRAD) specimens as compared with those in normal tissue specimens, which datasets were obtained from The Cancer Genome Atlas (TCGA) public PRAD and The Genotype‐Tissue Expression (GTEx) public normal prostate tissues (Fig. 1A). Consistently, *BAP1* mRNA levels were lower in tumor tissue than those in normal tissue (Fig. 1B), and higher in early‐stage PCa tissues with Gleason score 6 than those in late‐stage PCa tissue with Gleason score 7/8) (Fig. 1C), which data were obtained from R2 database (https://hgserver1.amc.nl/cgi‐bin/r2/main.cgi). Furthermore, BAP1 protein levels in PCa specimens from The Human Protein Atlas database (http://www.proteinatlas.org/) were analyzed to show that BAP1 protein levels were obviously reduced in the high‐grade specimens compared to those in the low‐ and medium‐grade tissues (Fig. 1D,E). In addition, the translocation of BAP1 was observed during tumor progression. We found that more BAP1 protein located in the cytoplasm or membranous fraction in the low/medium‐grade specimens, whereas BAP1 mainly located in the nucleus fraction in the high‐grade specimens (Fig. 1D), indicating that the localization of BAP1 in the cytoplasm or membranous may be beneficial to its tumor‐suppressive function. Our previous studies demonstrated that P69/M12 cell lines can be used as an experimental model for the study of PCa progression [44, 48]. P69 is a low‐tumorigenic and non‐metastatic prostate epithelial cell line whereas M12 is a highly tumorigenic and metastatic subline derived from P69 by selection in nude mice [45]. To find out the potential role of BAP1 in PCa progression, the protein levels of BAP1 in P69 and M12 cells were detected. The result showed that BAP1 was greatly downregulated in M12 cells compared with that in P69 (Fig. 1F). Moreover, we found a uniform reduced BAP1 protein levels in renal carcinoma cell lines (Fig. S1A), lung cancer cell lines (Fig. S1B), and breast cancer cell lines (Fig. S1C) in comparison with the normal renal cells (HK‐2 cells), lung cells (WI38 and 16HBE cells), and breast cells (MCF10A cells), respectively. Thus, above data suggested that loss or low expression of BAP1 might be an important event in tumorigenesis and progression.

### 3.2 BAP1 suppresses PCa cell progression

**Fig. 2:**
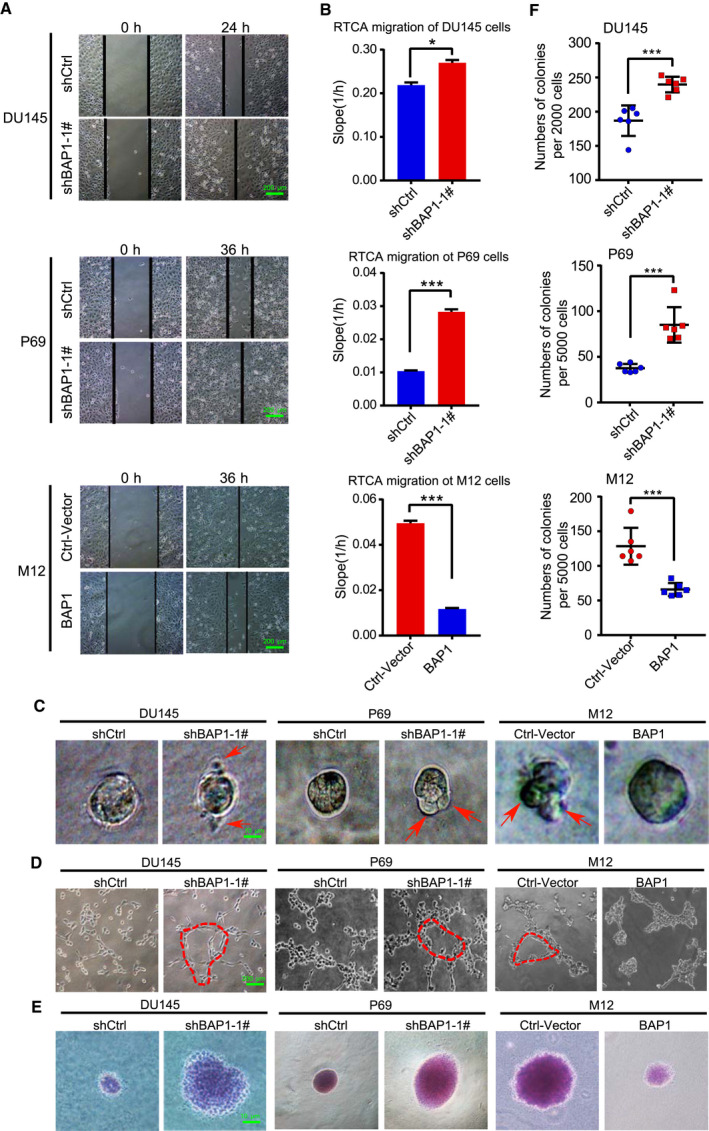
BAP1 suppresses PCa cell progression. (A) Wound‐healing assays for migration analysis of DU145, P69, and M12 stable cell lines. Representative pictures were taken at indicated times. Scale bars: 200 μm. (B) RTCA‐migration assays by using the xCELLigence RTCA‐DP system (*n* = 3). DU145, P69, and M12 stable cell lines were seeded into a CIM plate and subjected to a dynamic migration assay, respectively. The slope was shown as histogram. (C) 3D cell culture assays for DU145, P69 and M12 stable cell lines. The representative photographs of cell morphology were taken at 4 days. Scale bars: 10 μm. (D) Vasculogenic mimicry assays for DU145, P69 and M12 stable cells. The representative photographs of VM were taken with microscope 20 h later. Scale bars: 200 μm. (E, F) Soft‐agar colony formation assays for DU145, P69, and M12 stable cell lines (*n* = 6). Scale bars: 10 μm. The representative photographs of colonies were taken (E), and the number of colonies was scored (F). The DU145 and P69 stable cells used in (A–F) were established by using BAP1‐shRNA‐1#. Error bars in B and F indicated mean ± SD. Data analysis in B and F was conducted by unpaired t‐test (*P < 0.05, **P < 0.01, ***P < 0.001).

To investigate the potential role of BAP1 in PCa progression, BAP1 was silenced by shRNAs targeting the 3′‐terminal untranslated region (UTR) of *BAP1* mRNA in DU145 and P69 cells (Fig. S2A), or ectopically expressed in M12 cells by the lentiviral expressing system. Wound‐healing (Fig. 2A and Fig. S2B) and RTCA (real‐time cell analysis) assays (Fig. 2B and Fig. S2C) were conducted to evaluate the effects of BAP1 on migration, showing that cell migratory capacity was enhanced by silencing of BAP1 in DU145 and P69 cells whereas drastically decreased by ectopic expression of BAP1 in M12 cells. The 3D culture growth assay and vasculogenic mimicry (VM) formation assay were performed to assess the effects of BAP1 on cell aggressiveness according to our previous protocol as described [49]. 3D culture growth analysis showed that the knockdown of BAP1 in DU145 and P69 stable cells displayed scattered and invasive morphology compared to the cells with control shRNA (shCtrl), whereas ectopic expression of BAP1 in M12 cells abolished the ability of cell invading into the Matrigel but instead grew into tight colonies (Fig. 2C and Fig. S3A). In consistent with these results, the formation of vascular‐like shape was strongly induced by knockdown of BAP1 in DU145 and P69 cells while reduced by overexpression of BAP1 in M12 cells (Fig. 2D and Fig. S3B). Very similarly, the results of soft‐agar colony formation assays showed that silencing of BAP1 in DU145 and P69 cells massively increased while ectopic expression of BAP1 in M12 cells decreased colony formation both in numbers and sizes (Fig. 2E‐F and Fig. S3C,D). Taken together, above results demonstrated that BAP1 played tumor‐suppressive roles in PCa cell progression through inhibiting cell migration, invasion, and anchorage‐independent growth.

### 3.3 BAP1 suppresses PI3K‐Akt pathway by stabilizing PTEN

**Fig. 3:**
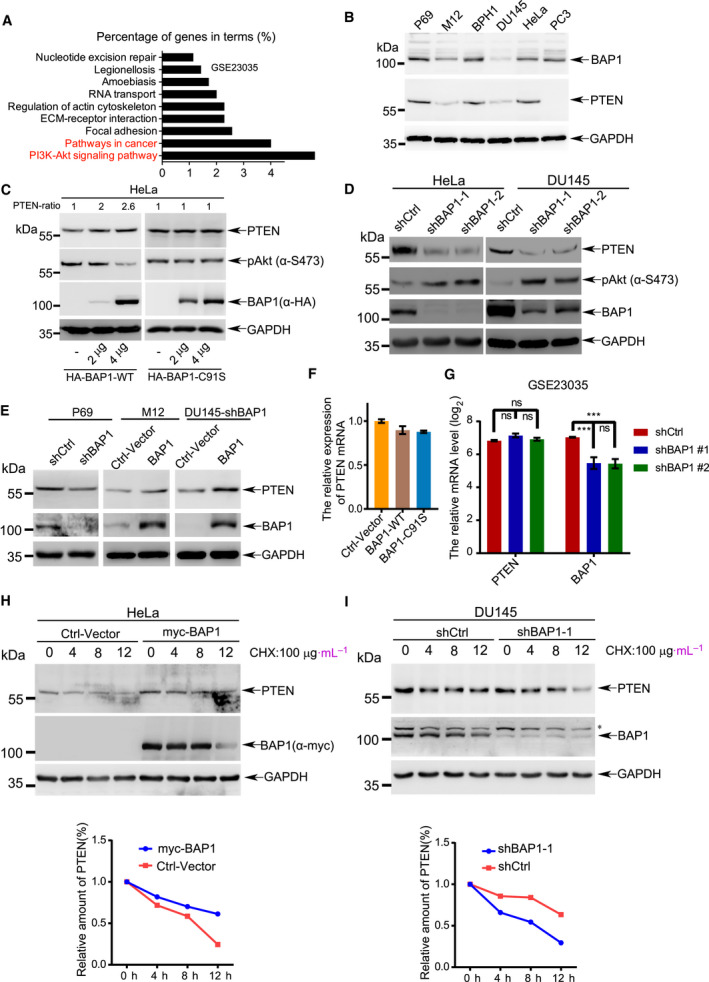
BAP1 suppresses PI3K‐Akt pathway by stabilizing PTEN. (A) The differentially expressed genes (fold change: 2, adjusted *P*‐value: 0.05) from GEO dataset (accession ID: GSE23035) were subjected to KEGG pathway enrichment analysis by DAVID. (B) The protein levels of BAP1 in cancer cell lines P69, M12, DU145, BPH1, PC3, and HeLa were analyzed by western blotting. (C) Western blotting analysis for endogenous PTEN and p‐Akt (Ser473) in HeLa cells transfected with HA‐BAP1^WT^ or HA‐BAP1^C91S^. PTEN bands were quantified by ImageJ software. (D) Western blotting analysis for endogenous PTEN, BAP1, and p‐Akt (Ser473) in DU145 and HeLa stable cells with BAP1 knockdown. (E) Western blotting analysis for endogenous PTEN and BAP1 in the indicated PCa stable cell lines. (F) The mRNA levels of PTEN in HeLa cells transfected with BAP1^WT^ or BAP1^C91S^ were analyzed by qRT–PCR. (G) The mRNA levels of PTEN in U2OS cells from GEO database (GSE23035) were analyzed by Limma package. (H, I) The half‐life of endogenous PTEN protein was determined in BAP1 overexpressed HeLa cells (H) and BAP1‐knockdown DU145 cells (I) with treatment of CHX (100 μg·mL^−1^) for the indicated time points. The asterisk indicated nonspecific band. All above experiments were repeated at least 3 times, and representative images were shown.

To investigate the potential mechanism underlying BAP1 in the regulation of tumor suppression, we analyzed the gene expression profile of *BAP1*‐knockdown U2OS cells, which was obtained from the Gene Expression Omnibus (GEO) public microarray dataset (accession ID: GSE23035) [28]. Differentially expressed genes (fold change ≥ 2, FDR < 0.05) were selected and then subjected to the KEGG pathway enrichment analysis by online tool DAVID (http://david.ncifcrf.gov/home.jsp), which showed that the differentially expressed genes were enriched for tumor‐associated pathways such as PI3K‐Akt signaling pathway and pathways in cancer (Fig. 3A). Among these pathways, BAP1 had the most significant effect on PI3K‐Akt signaling pathway, suggesting that BAP1 suppresses tumorigenesis by regulating key targets involved in the PI3K‐Akt pathway. In order to identify the potential targets of BAP1, lysates from prostate cancer cells P69, M12, DU145, PC3, and BPH1 (a benign prostatic hyperplasia epithelial cell line) as well as HeLa cells were used for western blotting analysis, showing that the protein levels of PTEN, which is a crucial regulator for the PI3K‐Akt pathway, were positively correlated with BAP1, except that in PC3 cells which is lack of PTEN expression [42] (Fig. 3B).

PTEN is one of the most important tumor suppressors and plays important roles in tumorigenesis by antagonizing PI3K/Akt signaling [42, 50]. To test whether PTEN protein is a potential target of BAP1, we transiently expressed BAP1‐WT or BAP1‐C^91^S in HeLa cells. The results showed that PTEN protein levels were significantly increased and sequentially the phosphorylation levels of Akt (pSer473) were decreased (Fig. 3C, left panel), while BAP1 enzyme‐dead mutant (BAP1‐C^91^S) had no effect (Fig. 3C, right panel). In line with this, decreased protein levels of PTEN and increased phosphorylation levels of Akt were observed in stable knockdown of BAP1 in HeLa and DU145 cells by using the lentiviral shRNA system (Fig. 3D). Furthermore, silencing of endogenous BAP1 in P69 cells downregulated the PTEN protein levels; on the contrary, overexpression of BAP1 in M12 cells upregulated the PTEN protein levels (Fig. 3E). More importantly, re‐expression of BAP1 rescued the effect of PTEN downregulation in DU145‐shBAP1 cells (Fig. 3E, right panel). These data demonstrated that BAP1 increased/stabilized PTEN protein.

Since BAP1 is a deubiquitinating enzyme which is involved in transcriptional regulation through interacting with ASXL1/2, HCF1, YY1, FoxK1/K2, etc. [15]. To exclude the possibility that BAP1 affects PTEN expression on the transcriptional level, total RNAs from HeLa cells transiently expressing wild‐type BAP1 or enzymatically defective mutant BAP1^C91S^ were extracted for quantitative real‐time PCR (qRT–PCR). The results showed there were no significant changes in the PTEN mRNA levels (Fig. 3F), suggesting that BAP1 did not regulate PTEN expression at the transcriptional level. The similar results were also observed in GEO datasets GSE23035 [28] (Fig. 3G). Next to determine whether BAP1 influences the stability of PTEN protein, the half‐life of endogenous PTEN was measured by treatment of cycloheximide (CHX), an inhibitor of protein translation. The result showed that the half‐life of endogenous PTEN protein was prolonged in HeLa cells transfected with Myc‐tagged BAP1 compared with that in the control‐vector transfected cells (Fig. 3H). Conversely, the half‐life of PTEN protein was shortened by knockdown of BAP1 in DU145 cells (Fig. 3I). Collectively, these results suggested that BAP1 stabilized PTEN protein and thus downregulated the PI3K‐Akt signaling pathway.

### 3.4 BAP1 directly interacts with PTEN

**Fig. 4:**
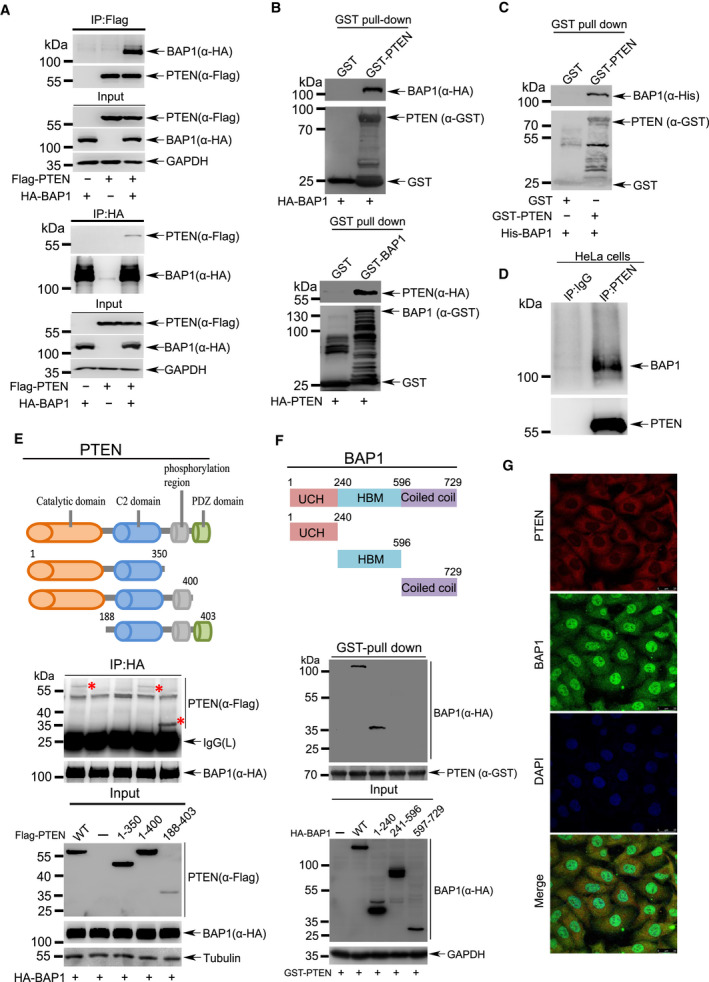
BAP1 directly interacts with PTEN. (A) Lysates from 293T cells transfected with Flag‐PTEN or/and HA‐BAP1 were immunoprecipitated with anti‐Flag antibody, and then immunoblotted with anti‐HA antibody. The reciprocal immunoprecipitation was also conducted to validate the interaction. (B) Purified GST‐PTEN or GST‐BAP1 was incubated with lysates from 293T cells expressing HA‐BAP1 or HA‐PTEN, respectively, for pull‐down assay of protein–protein interaction. (C) GST‐fusion protein pull‐down assay with purified GST‐PTEN and His‐BAP1. (D)The interaction between endogenous BAP1 and PTEN. Lysates from HeLa cells was used for Co‐IP with anti‐PTEN antibody, and followed by western blotting analysis with anti‐BAP1 antibody. Anti‐immunoglobulin (IgG) served as the negative control. (E) Upper panel, a series of truncated forms of Flag‐PTEN were generated based on its domains including a catalytic domain, a C2 domain, a multisite phosphorylation domain, and a PDZ‐binding domain. Middle panel, lysates from 293T cells transfected with HA‐BAP1 full‐length or truncated Flag‐PTEN were co‐immunoprecipitated with anti‐HA antibody, and followed by western blotting analysis with anti‐Flag antibody. Lower panel, immunoblotting was performed for Input. (F) Upper panel, a series of truncated forms of HA‐BAP1 were generated based on its domains including an ubiquitin C‐terminal hydrolase domain (UCH), a HCF‐binding motif (HBM), and a coiled‐coil domain with two nuclear localization sequences (NLS) at its C terminus. Middle panel, lysates from 293T cells transfected with full‐length or truncated HA‐BAP1 were incubated with bacterially produced GST‐PTEN for GST pull‐down assays *in vitro*. Lower panel, immunoblotting was performed for Input. (G) Colocalization of PTEN and BAP1 in DU145 cells stably expressing PTEN. Scale bars: 25 μm. All above experiments were repeated at least 3 times.

To determine whether BAP1 directly interacts with PTEN, 293T cells were cotransfected with Flag‐tagged PTEN and HA‐tagged BAP1. The reciprocal co‐immunoprecipitation (co‐IP) assays with anti‐Flag or anti‐HA antibody showed that BAP1 interacted with PTEN (Fig. 4A). GST pull‐down assays were performed by using bacterially expressed glutathione S‐transferase (GST)‐tagged PTEN or (GST)‐tagged BAP1 recombinant protein in incubation with lysates from 293T cells overexpressing HA‐tagged BAP1 or HA‐tagged PTEN, respectively, to confirm the interaction between BAP1 and PTEN (Fig. 4B). More confidently, GST‐PTEN was able to directly bind to His‐tagged BAP1 protein which was also purified from *E. coli* expressing system (Fig. 4C). Furthermore, we validated this direct interaction between endogenous BAP1 and PTEN in HeLa cells by Co‐IP/WB (Fig. 4D).

Next to map the BAP1‐binding region of PTEN, we generated a series of truncated forms based on domains of PTEN, which mainly consists of an aminoterminal phosphatase domain, a C2 domain, and a carboxy‐terminal PDZ motif. The truncated PTEN forms as indicated and along with HA‐tagged BAP1 were transfected into 293T cells. The results of co‐IP/WB revealed that BAP1 could efficiently bind to full‐length PTEN, PTEN (1–400), and PTEN (188–403), except the truncated PTEN (1–350) (Fig. 4E), indicating that the BAP1‐binding domain of PTEN might be located in the region of 350–400 amino acid (aa) residues. Moreover, to determine which region of BAP1 is responsible for binding with PTEN, we further generated a series of truncated forms of BAP, which is composed of an ubiquitin C‐terminal hydrolase (UCH) domain, a HBM, and a coiled‐coil domain. GST‐PTEN was incubated with lysates from 293T cells transfected with HA‐BAP1(WT) or three HA‐tagged truncated BAP1 for GST pull‐down assays. The result showed that both the UCH domain (1–240 aa) and the full‐length BAP1 could specifically bind with GST‐PTEN (Fig. 4F), suggesting that the UCH domain of BAP1 was essential for its physical interaction with PTEN. Taken together, these results demonstrated that BAP1 directly interacted with PTEN *in vitro* and *in vivo*.

To determine whether BAP1‐PTEN interaction occurs in the cytoplasm or nucleus, we analyzed subcellular fractionations from BPH1, DU145, P69, and M12 cells. The results showed that BAP1 proteins were present both in the nucleus and in the cytoplasm, while PTEN proteins in the cytoplasm were much more than those in the nucleus (Fig. S4A). By using immunofluorescence staining, we confirmed that PTEN colocalized with endogenous or ectopically expressed BAP1 in the cytoplasm of DU145 stable cells (Fig. 4G) and HeLa cells (Fig. S4B), respectively. In addition, the deletion of nuclear localization sequence (ΔNLS) abolished the localization of BAP1 in the nucleus (Fig. S4C), and this form BAP1^ΔNLS^ had same ability as BAP^WT^ in interacting with and stabilizing PTEN (Fig. S4D,E), which further suggested that BAP1 bound to PTEN in the cytoplasm.

### 3.5 BAP1 deubiquitinates PTEN

**Fig. 5:**
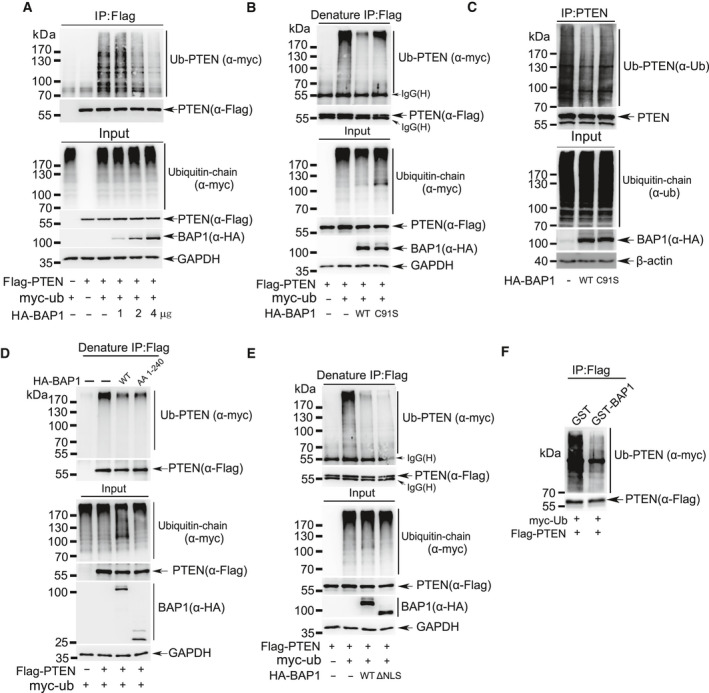
BAP1 deubiquitinates PTEN. (A) Lysates from 293T cells transfected with Flag‐PTEN, Myc‐Ubiquitin together with an increasing amount of BAP1 were immunoprecipitated with anti‐Flag antibody, and followed by western blotting analysis with anti‐Myc antibody. (B) Lysates from 293T cells transfected with Flag‐PTEN, Myc‐Ubiquitin together with BAP1^WT^ or mutant BAP1^C91S^ were immunoprecipitated with anti‐Flag antibody under denaturing condition, and followed by western blotting analysis with anti‐Myc antibody. (C) Lysates from HeLa cells transfected with wild‐type BAP1 or mutant BAP1^C91S^ were immunoprecipitated with anti‐PTEN antibody, and followed by western blotting analysis with anti‐ubiquitin antibody. (D‐E) Lysates from 293T cells transfected with Flag‐PTEN, Myc‐Ubiquitin together with full‐length HA‐BAP1, truncated HA‐BAP1^1‐240^ (D) or HA‐BAP1^ΔNLS^ (E) were immunoprecipitated with anti‐Flag antibody under denaturing condition, and followed by western blotting analysis with anti‐Myc antibody. (F) Ubiquitinated PTEN was purified from 293T cells transfected with Flag‐PTEN and Myc‐Ubiquitin, and then incubated with purified GST‐BAP1 from *E. coli*. PTEN ubiquitination level was detected by western blotting with anti‐Myc antibody. All above experiments were repeated at least 3 times.

Since above results have proven that BAP1 directly interacted with and stabilized PTEN protein, and BAP1 is a member of the ubiquitin C‐terminal hydrolase DUB subfamily, we speculated that BAP1 might remove polyubiquitin chains from PTEN. To validate this assumption, we performed immunoprecipitation assays under natural or denaturing condition to examine PTEN ubiquitination in the presence of BAP1. Plasmids Flag‐PTEN and Myc‐Ub along with an increasing amount of BAP1 were cotransfected into 293T cells. Cell lysates were harvested for IP and western blotting analysis, showing that polyubiquitination of PTEN was gradually reduced by BAP1 in a dose‐dependent manner (Fig. 5A and Fig. S5A). Next, plasmids Flag‐PTEN and Myc‐Ub along with BAP1^WT^ or inactive mutant BAP1^C91S^ were cotransfected into 293T cells. The results showed that polyubiquitination of PTEN was markedly decreased by coexpression of BAP1^WT^ but not BAP1^C91S^ (Fig. 5B and Fig. S5B). Further, only BAP1^WT^ or BAP1^C91S^ was transfected. The similar result that deubiquitination of endogenous PTEN occurred in overexpression of BAP1^WT^ rather than BAP1^C91S^ in HeLa cells was observed (Fig. 5C). These results revealed that ubiquitination of PTEN was specifically reduced by BAP1, whose enzymatic activity was indispensable.

As we have shown that the truncated form BAP1^1‐240^ containing a UCH domain directly interacted with PTEN (Fig. 4F), we wondered that this truncated form of BAP1 is enough and responsible for deubiquitination of PTEN. Flag‐PTEN and Myc‐Ub together with BAP1^WT^ or BAP1^1‐240^ were transfected into 293T cells, respectively. The results of IP/WB analysis revealed that BAP1^1‐240^ was sufficient to deubiquitinate PTEN (Fig. 5D and Fig. S5C). Furthermore, to detected whether recombinant GST‐BAP1 can directly remove polyubiquitination of PTEN *in vitro,* polyubiquitinated PTEN protein by immunoprecipitation from 293T cells overexpressing Flag‐PTEN and Myc‐Ub was incubated with bacterially produced recombinant protein GST‐BAP1 in a cell‐free system. As expectedly, GST‐BAP1 significantly removed polyubiquitination of PTEN *in vitro* (Fig. 5E). Collectively, these results suggested that BAP1 was a specific deubiquitinase for PTEN.

### 3.6 Suppression of tumorigenesis by BAP1 depends on PTEN

As an important tumor suppresser, tiny changes in PTEN protein levels have a big impact on tumorigenesis [42, 51, 52, 53]. To better understand the importance of PTEN in PCa progression, we analyzed the effect of PTEN protein levels on Akt signaling pathway and soft‐agar colony formation. As expectedly, we found that knockdown of PTEN in DU145 cells increased whereas overexpression of PTEN reduced phosphorylation levels of Akt (pSer473) (Fig. S6A). Consistently, the soft‐agar colony formation assay showed that knockdown of PTEN massively increased DU145 cell colony formation, while overexpression of PTEN significantly decreased it (Fig. S6B,C). These results validated that that PTEN suppressed anchorage‐independent cell growth of DU145 by inactivation of Akt pathway.

**Fig. 6:**
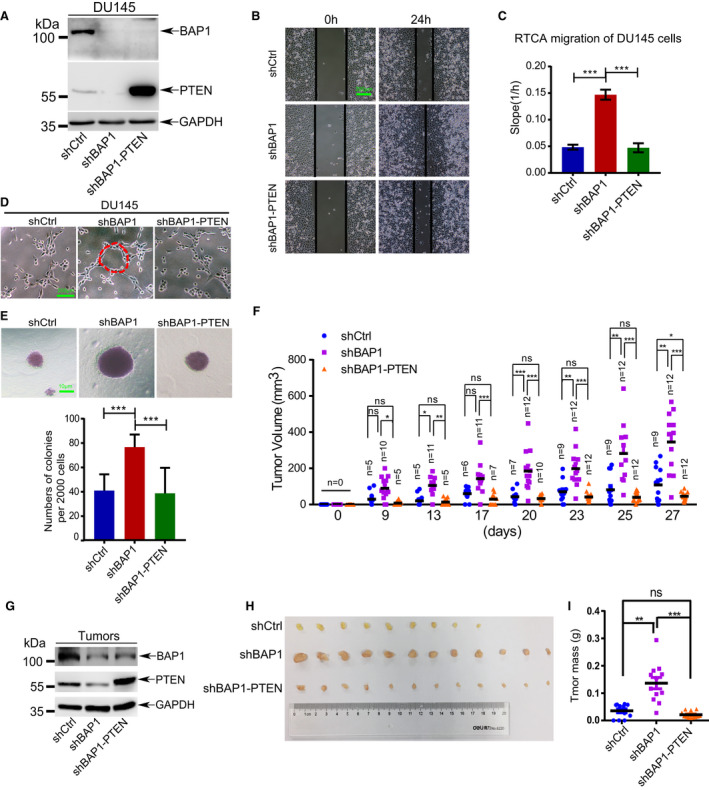
Suppression of tumorigenesis by BAP1 is dependent on PTEN. (A) Western blotting analysis for BAP1 and PTEN in the stable DU145 cell lines, and GAPDH was served as loading control. (B) Wound‐healing assays for migration analysis of the indicated DU145 stable cells. Scale bars: 200 μm. (C) RTCA‐migration assays of DU145 stable cells by using the xCELLigence RTCA‐DP system (*n* = 3). The slope was shown as histogram. (D) Vasculogenic mimicry assays for the indicated stable cells. The representative photographs were taken. Scale bars: 200 μm. (E) Soft‐agar colony formation assay of DU145 stable cells (*n* = 6). The representative photographs of colonies were taken (upper panel), and the number of colonies was scored (lower panel). Scale bars: 10 μm. (F) Stable DU145 cells were injected subcutaneously into male BALB/c nude mice, the volume of tumors was measured at the indicated times after injection (*n* = number of tumors in each group). (G) Western blotting analysis for BAP1 and PTEN in tumor tissues. GAPDH was used as loading control. (H, I) Xenograft tumors were dissected (H), and weight was assessed (I). Error bars in C, E, and I indicated mean ± SD. Data analysis in C, E, and I was conducted by unpaired t‐test (*P < 0.05, **P < 0.01, ***P < 0.001).

To verify whether inhibition of tumorigenesis by BAP1 is dependent on PTEN, we stably reintroduced PTEN into a BAP1‐knockdown DU145 cell line (DU145‐shBAP1‐1#) (Fig. 6A) and carried out a series of rescue experiments. Overexpression of PTEN in DU145‐shBAP1‐1# cells abolished the enhanced cell migration (Fig. 6B,C and Fig. S7A), VM formation (Fig. 6D) and cell anchorage‐independent growth (Fig. 6E and Fig. S7B) induced by BAP1 silencing, which suggested that BAP1 repressed tumorigenesis in a PTEN‐dependent way. Furthermore, to investigate the functional correlation between BAP1 and PTEN *in vivo*, xenografted tumor growth analysis was performed. The same DU145 stable cell lines were subcutaneously injected into the back of male BALB/c nude mice, and tumor growth was monitored. As expectedly, xenografted tumor growth was markedly increased by knockdown of endogenous BAP1, which was reversely rescued by re‐expression of PTEN (Fig. 6F). The expression levels of both BAP1 and PTEN in xenograft tumors were validated by western blotting analysis (Fig. 6G). The photographs were taken, and weight of tumors was analyzed after killing the nude mice at 27 days after injection, showing significant increases in sizes (Fig. 6H) and weights (Fig. 6I) of xenograft tumors of DU145‐shBAP1‐1# cells. Notably, restoring PTEN expression could completely reverse the tumor‐promoting effect of BAP1 knockdown *in vivo*. To further determine whether BAP1 function of tumor suppression is dependent on inhibiting the Akt signaling pathway, we silenced Akt1 by lenti‐shRNAs in DU145‐shBAP1‐1# cells (Fig. S8A). BAP1/Akt1 double knockdown of DU145 cells led to a reduction on anchorage‐independent cell growth (Fig. S8B,C) and cell aggressiveness (Fig. S8D,E) in compared with only BAP1 knockdown, suggesting that silencing of Akt1 rescued from effects caused by BAP1 depletion. Taken together, BAP1 contributed to tumor suppressor by stabilizing PTEN to suppress Akt activation.

### 3.7 The correlation of BAP1 and PTEN in clinical PCa specimens

**Fig. 7:**
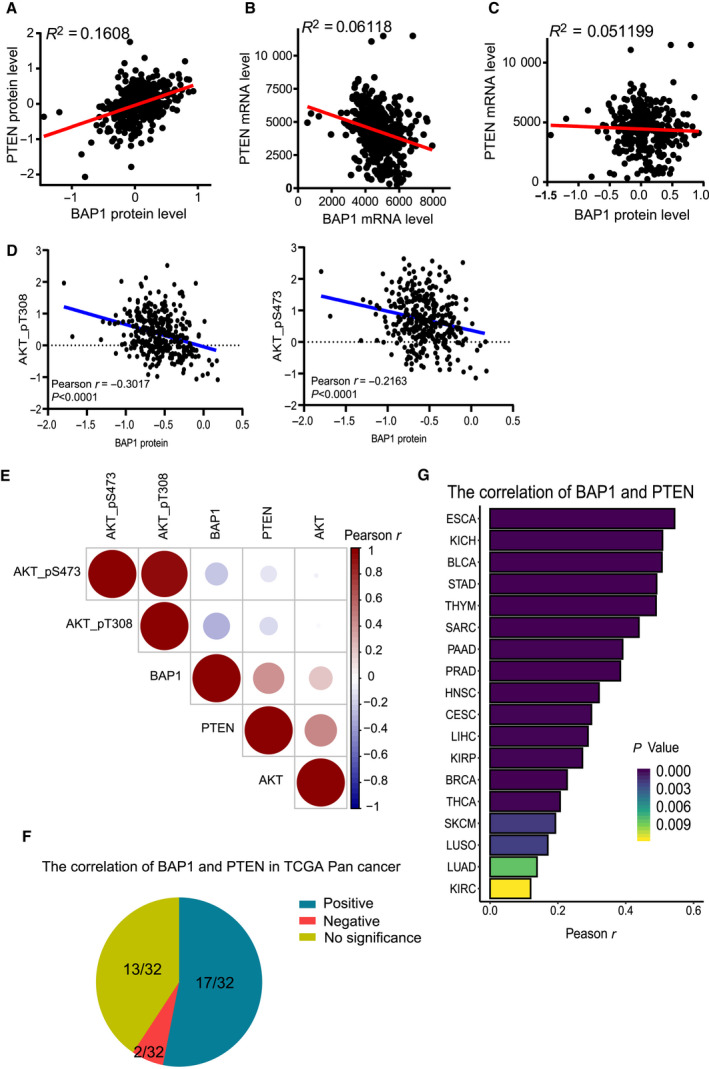
The correlation of BAP1 and PTEN in clinical PCa specimens. (A) The correlation between the protein levels of BAP1 and PTEN in clinical PCa specimens (*n* = 351) from TCGA database was analyzed by linear regression. (B) The correlation between the mRNA levels of *BAP1* and *PTEN* in clinical specimens from TCGA database was analyzed by linear regression. (C) The correlation between BAP1 protein and *PTEN* mRNA in clinical specimens from TCGA database was analyzed by linear regression. (D) The correlation between the levels of BAP1 protein and AKT_pT308 (left), or AKT_pS473 (right) in clinical PCa specimens from TCGA database were analyzed by Pearson correlation test and linear regression. (E)The correlation between the levels of BAP1 protein, PTEN protein, AKT protein, AKT_pT308, and AKT_pS473 (right) in clinical PCa specimens from TCGA database was analyzed by Pearson correlation test. (F) The correlation between the protein levels of BAP1 and PTEN in 32 kinds of human cancers from TCGA database. (G) The histogram of human cancers in which the protein levels of BAP1 and PTEN are positively related.

Next, we investigated clinical relevance by analyzing the expression levels of BAP1 and PTEN in clinical prostate cancer tissues. We analyzed the protein levels of BAP1 and PTEN in PCa tissues (*n* = 351) from the TCGA database (The Cancer Genome Atlas) to reveal significant positive correlation between BAP1 and PTEN (linear regression, *R* ^2^ = 0.1608, *P* < 0.0001) (Fig. 7A). However, the positive correlation was not observed between *BAP1* mRNAs and *PTEN* mRNAs (linear regression, *R* ^2^ = 0.06118) (Fig. 7B), or between BAP1 proteins and *PTEN* mRNAs (linear regression, *R* ^2^ = 0.001599) (Fig. 7C), which was consistent with our previous results that BAP1 protein stabilized PTEN by direct interaction with each other for deubiquitination of PTEN. Moreover, the BAP1 protein levels were negatively correlated with AKT_pT308 and AKT_pS473 (Fig. 7D). Collectively, BAP1 protein was positively correlated with PTEN protein, whereas both BAP1 and PTEN proteins showed negative associations with AKT_pT308 and AKT_pS473 in clinical PCa specimens (Fig. 7E), revealing that BAP1 made pivotal contribution to the PI3K/Akt signaling pathway through regulating PTEN in PCa progression.

Finally, we also extended our study to a variety of cancers besides PCa. The protein data including 32 kinds of cancers from TCGA (listed in Table S2) were collected for analysis, showing general positive correlations between BAP1 and PTEN in 17 of them, including esophageal carcinoma (ESCA), kidney chromophobe (KICH), bladder urothelial carcinoma (BLCA), and prostate adenocarcinoma (PRAD), whereas slight negative correlations only in brain lower grade glioma (LGG) and testicular germ cell tumors (TGCT) (Fig. 7F,G). These data indicated that BAP1 was an essential regulator of PTEN in many kinds of human cancers, which suggested that the BAP1‐PTEN signaling axis plays important roles in tumor suppression.

## 4 Discussion

It has been well‐established that BAP1 is an important tumor suppressor and frequently mutated in many human cancers. However, the role of BAP1 in PCa is still unclear. In this study, we revealed that BAP1 suppressed PCa progression through deubiquitinating and stabilizing PTEN. More importantly, large‐scale clinical relevance analyses showed that BAP1 was positively related to PTEN at the protein level in many kinds of human cancers (Fig. 7), suggesting that the positive correlation between BAP1 and PTEN may be ubiquitous.

PTEN is a major tumor suppressor and frequently mutated or deleted in various human cancers. PTEN has been well documented to attenuate cancer cell cycle progression, migration, invasion, and tumorigenesis by antagonizing the PI3K/AKT signaling pathway [50, 54]. Homozygous deletion of *Pten* in mice results in early embryonic lethality, whereas heterozygous *Pten* in mice leads to multiple tumors [55, 56, 57]. PTEN alteration is strongly responsible for PCa development. The *PTEN* prostate‐specific knockout mice show a significant shortened latency of PIN (prostatic intraepithelial neoplasia) formation and metastatic PCa, which are similar with the progression seen in human prostate disease [58]. Dysregulation of PTEN results in tumor initiation and progression with PCa being one of the most sensitive cancers [53].

In addition to *PTEN* gene mutations and deletions, recent studies have revealed the importance of post‐translational modifications, especially ubiquitination, in the regulation of PTEN stability, activity, and localization. Ubiquitin E3 ligases including NEDD4‐1 [52], XIAP [59], WWP2 [60], CHIP [61], TRIM27 [62], SPOP [63], MKRN1 [64], CRL4B [65], and FBXO22 [66] for PTEN ubiquitination have been identified. On the contrary, the regulation of PTEN deubiquitination remains relatively poorly understood. Several deubiquitinases (DUBs) for PTEN have been reported. USP7/HAUSP can remove the monoubiquitination of PTEN and induce its nuclear export in acute promyelocytic leukemia [67]. Through proteome‐wide screen of DUB members, USP13 [51] and OTUD3 [68] have been identified as deubiquitinases which directly bind to and stabilize PTEN in breast cancer. However, depletion of USP13 or OTUD3 in cells reduces but does not completely abolish the PTEN ubiquitination, suggesting that other deubiquitinases of PTEN may exist. Intriguingly, here we identified that BAP1 served as a novel deubiquitinase of PTEN to control the PI3K‐Akt signaling pathway and cancer progression in PCa. Although it has been reported that somatic mutation of BAP1 is rare in PCa [69], the mRNAs and protein levels of BAP1 were significantly reduced in PCa tissues as compared with adjacent tissues (Fig. 1), which indicates there is a new mechanism underlying PCa progression. Notably, we observed that BAP1 knockdown contributed to PCa cells migration, invasion, transformation, and tumor growth (Fig. 2 and Figs S2 and S3). For the mechanism that BAP1 contributes to PCa suppression, we found that BAP1 deubiquitinated and stabilized PTEN (Fig. 5 and Fig. S5), thereby inhibiting activation of the Akt signaling pathway (Fig. 3). In addition, PTEN overexpression or Akt1 knockdown rescued the aggravated PCa progression caused by BAP1 silencing (Fig. 6 and Fig. S8). Taken together, our findings discovered a novel regulatory mechanism of BAP1‐PTEN‐PI3K/AKT by which BAP1 repressed PCa initiation and progression.

## 5 Conclusions

In summary, our findings demonstrate that BAP1 is an important deubiquitinase of PTEN for its stability and the BAP1‐PTEN signaling axis plays a crucial role in tumor suppression.

## Conflict of interest

The authors declare no conflict of interest.

## Author contributions

JY and RD conceived and designed the study. RD, XZ, YG, LL, and JH performed most of the experiments; ZQ, HZ, RC, and YW helped with all experiments; JY, XZ, and RD wrote the manuscript. All authors read and approved the final manuscript.

## Supporting information


**Fig. S1.** The protein level of BAP1 in renal carcinoma, lung cancer and breast cancer cell lines.

**Fig. S2.** BAP1 suppresses PCa cell migration.

**Fig. S3.** BAP1 suppresses PCa progression.

**Fig. S4.** PTEN interacts with BAP1 in the cytoplasm.

**Fig. S5.** BAP1 deubiquitinates PTEN.

**Fig. S6.** Downregulated PTEN expression promotes activation of Akt pathway and PCa cell colony formation.

**Fig. S7.** BAP1 inhibits tumorigenesis through maintenance of PTEN.

**Fig. S8.** BAP1 suppresses PCa progression in an Akt‐dependent manner.

**Table S1.** List of DNA or RNA oligonucleotides.
**Table S2.** The list of clinical cancers used for correlation analysis.

## Data Availability

All data required to evaluate the conclusions of the paper are present in the main text or the Supplementary Materials of the paper.
