# Supplementary material for: BAP1 suppresses prostate cancer progression by deubiquitinating and stabilizing PTEN
Source: Mol Oncol. 2020 Nov 20;15(1):279–98. doi: 10.1002/1878-0261.12844 (PMC7782096; doi:10.1002/1878-0261.12844)
Supplement: Supplementary file 1 — Fig. S1. The protein level of BAP1 in renal carcinoma, lung cancer and breast cancer cell lines. [file MOL2-15-279-s001.pdf]

Deng_Fig.S1

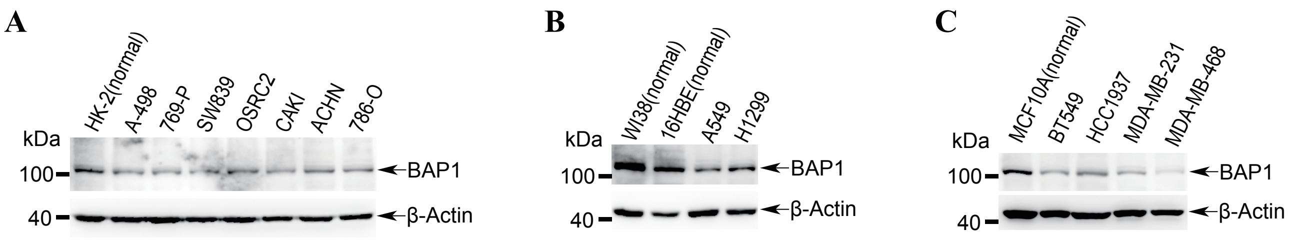

Fig. S1. The protein level of BAP1 in renal carcinoma cell lines (A), lung cancer cell lines (B) and breast cancer cell lines (C). Whole cell lysates were used for Western blotting with antibodies against BAP1 and β-actin.
