# Supplementary material for: BAP1 suppresses prostate cancer progression by deubiquitinating and stabilizing PTEN
Source: Mol Oncol. 2020 Nov 20;15(1):279–98. doi: 10.1002/1878-0261.12844 (PMC7782096; doi:10.1002/1878-0261.12844)
Supplement: Supplementary file 2 — Fig. S2. BAP1 suppresses PCa cell migration. [file MOL2-15-279-s002.pdf]

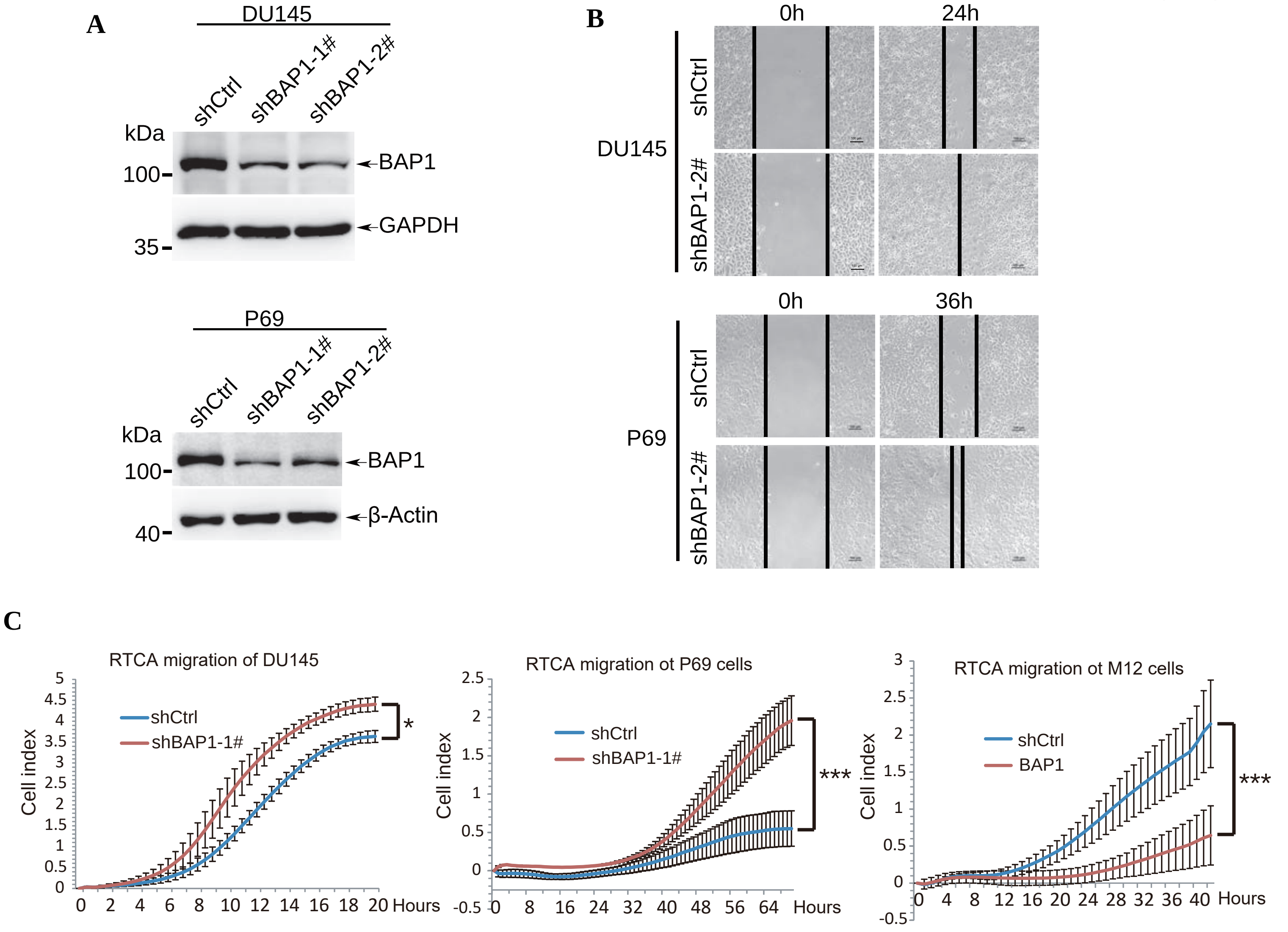

Fig. S2. BAP1 suppresses PCa cell migration. (A) The efficiency of BAP1 knockdown in DU145 and P69 cells used in Fig. 2, Fig. S2 and Fig.S3. BAP1 protein levels were determined by Western blotting analysis. GAPDH and β-Actin were used as internal controls. (B) Wound healing assays for DU145 and P69 stable cell lines with BAP1 knockdown using BAP1-shRNA-2#. Scale bars:100 μm. Representative pictures were taken at indicated times. (C) Stable DU145, P69 and M12 cells were serum starved for 4-6 hours, then detached with trypsin and resuspended in serum-free medium with concentration of 1×10^5 cells per ml. 100 μl of suspension was seeded into the pre-equilibrated upper chamber of the CIM-plate along with the bottom well containing complete medium for migration. Cell index values were detected every 15 min. Error bars indicated mean ±SD. These are related to Fig. 2B.
