# Supplementary material for: BAP1 suppresses prostate cancer progression by deubiquitinating and stabilizing PTEN
Source: Mol Oncol. 2020 Nov 20;15(1):279–98. doi: 10.1002/1878-0261.12844 (PMC7782096; doi:10.1002/1878-0261.12844)
Supplement: Supplementary file 3 — Fig. S3. BAP1 suppresses PCa progression. [file MOL2-15-279-s003.pdf]

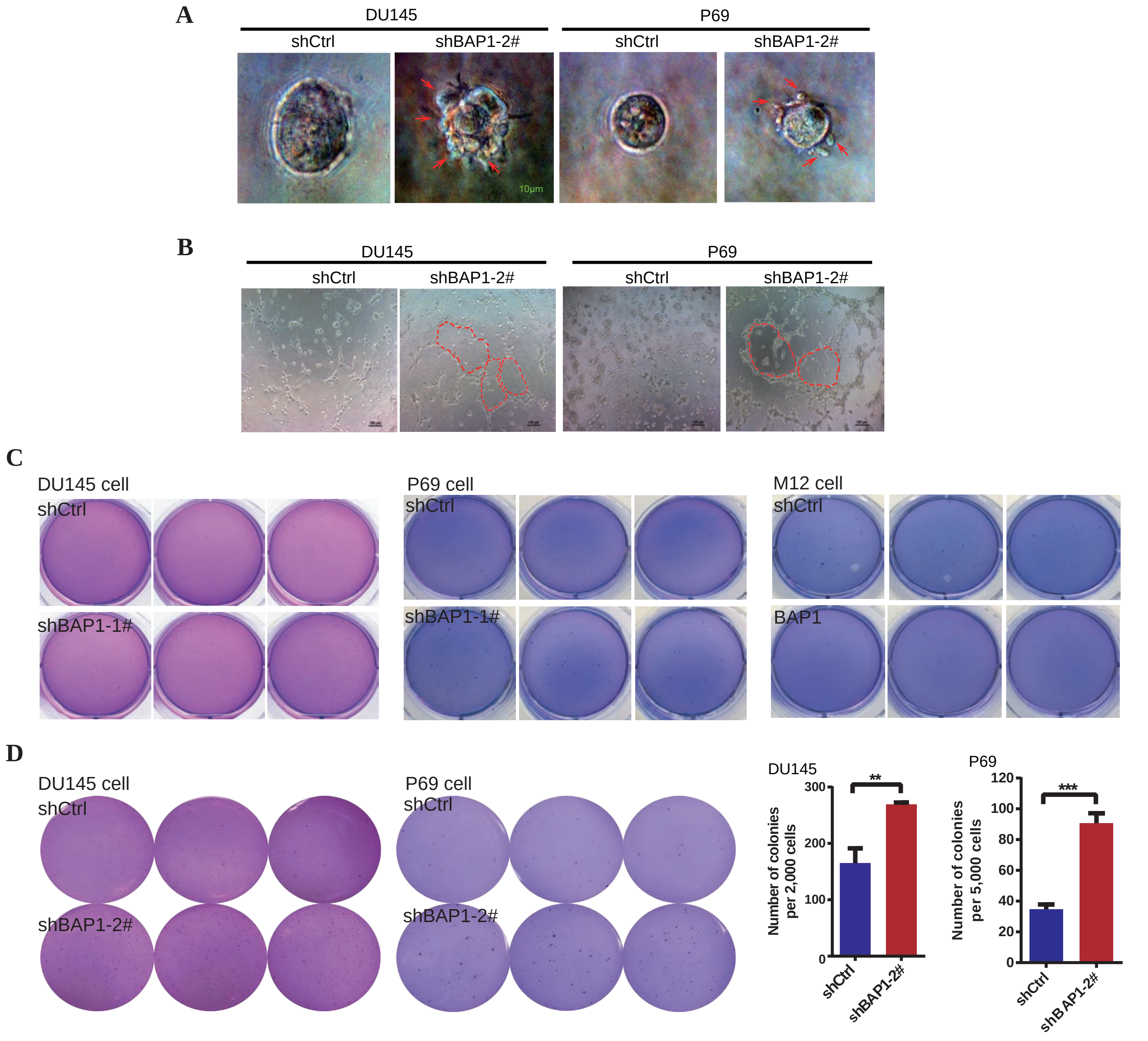

Fig. S3. BAP1 suppresses PCa progression. (A) 3D cell culture assays for DU145 and P69 stable cell lines with BAP1 knockdown using BAP1-shRNA-2#. Scale bars: 10 μm.The representative photos of cell morphology were taken at 4 days. (B) Vasculogenic mimicry assays for DU145 and P69 stable cell lines with BAP1 knockdown using BAP1-shRNA-2#. Scale bars: 100 μm. Representative pictures were taken at indicated times. (C) Stable DU145, P69 and M12 cell lines were seeded in 2 ml of medium containing 5% FBS with 0.35% agar at 2000 cells/well for DU145, 5000 cells/well for P69/M12 cells. The representative photographs of colonies were taken. These are related to Fig. 2E-F. (D) Soft agar colony formation assays for DU145 and P69 stable cell lines with BAP1 knockdown using BAP1-shRNA-2#. The representative photographs of colonies were taken and the number of colonies was scored.
