# Supplementary material for: BAP1 suppresses prostate cancer progression by deubiquitinating and stabilizing PTEN
Source: Mol Oncol. 2020 Nov 20;15(1):279–98. doi: 10.1002/1878-0261.12844 (PMC7782096; doi:10.1002/1878-0261.12844)
Supplement: Supplementary file 4 — Fig. S4. PTEN interacts with BAP1 in the cytoplasm. [file MOL2-15-279-s004.pdf]

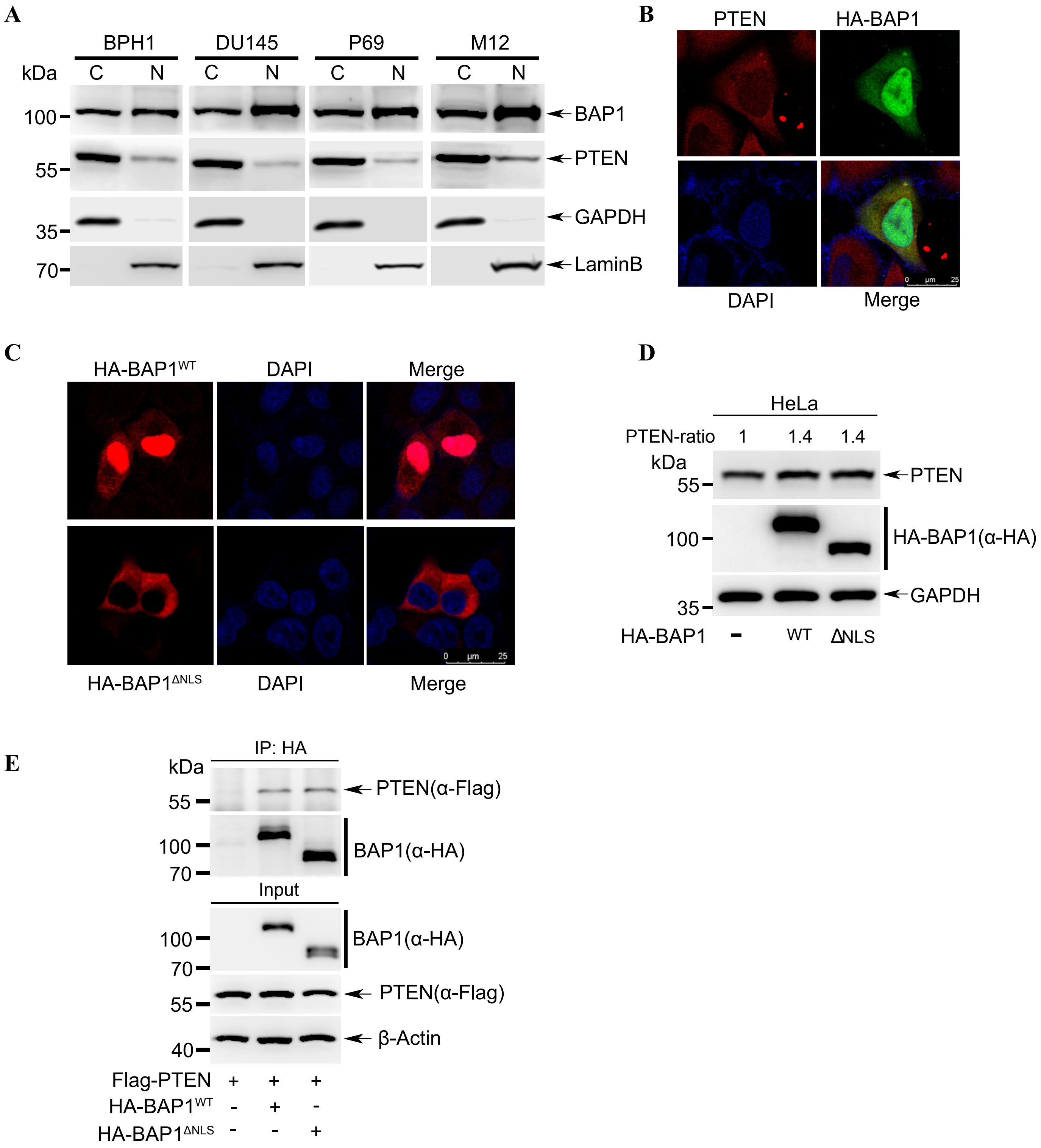

Fig. S4. PTEN interacts with BAP1 in the cytoplasm. (A) BAP1 resides in both the nucleus and cytoplasm. Cell extracts derived from BPH1, DU145, P69 and M12 cells were used to isolate subcellular fractionation, and then equal proportion from nuclear and cytoplasmic fractions was used for Western blotting analysis. C: Cytoplasm. N: Nucleus. (B) Colocalization of PTEN and BAP1 in cells. HA-BAP1 was transiently expressed in HeLa cells stably expressing PTEN. Immunofluorescence staining was performed and typical images captured using a confocal microscope (Leica TCS SP8) were shown. Scale bars: 25 μm. (C) The localization of $BAP1^{WT}$ and $BAP1^{\Delta NLS}$ in HeLa cells. $HA\text{-}BAP1^{WT}$ or $HA\text{-}BAP1^{\Delta NLS}$ was transiently expressed in HeLa cells. Immunofluorescence staining was performed and typical images captured using a confocal microscope (Leica TCS SP8) were shown. Nuclei were counterstained with DAPI (blue). Scale bars: 25 μm. (D) Western blotting analysis for endogenous PTEN in HeLa cells transfected with $HA\text{-}BAP1^{WT}$ or $HA\text{-}BAP1^{\Delta NLS}$. (E) Lysates from 293T cells transfected with Flag-PTEN and $HA\text{-}BAP1^{WT}$ or $HA\text{-}BAP1^{\Delta NLS}$ were immunoprecipitated with anti-HA antibody, and followed by Western blotting analysis with indicated antibodies.
