# Supplementary material for: BAP1 suppresses prostate cancer progression by deubiquitinating and stabilizing PTEN
Source: Mol Oncol. 2020 Nov 20;15(1):279–98. doi: 10.1002/1878-0261.12844 (PMC7782096; doi:10.1002/1878-0261.12844)
Supplement: Supplementary file 5 — Fig. S5. BAP1 deubiquitinates PTEN. [file MOL2-15-279-s005.pdf]

Deng_Fig.S5

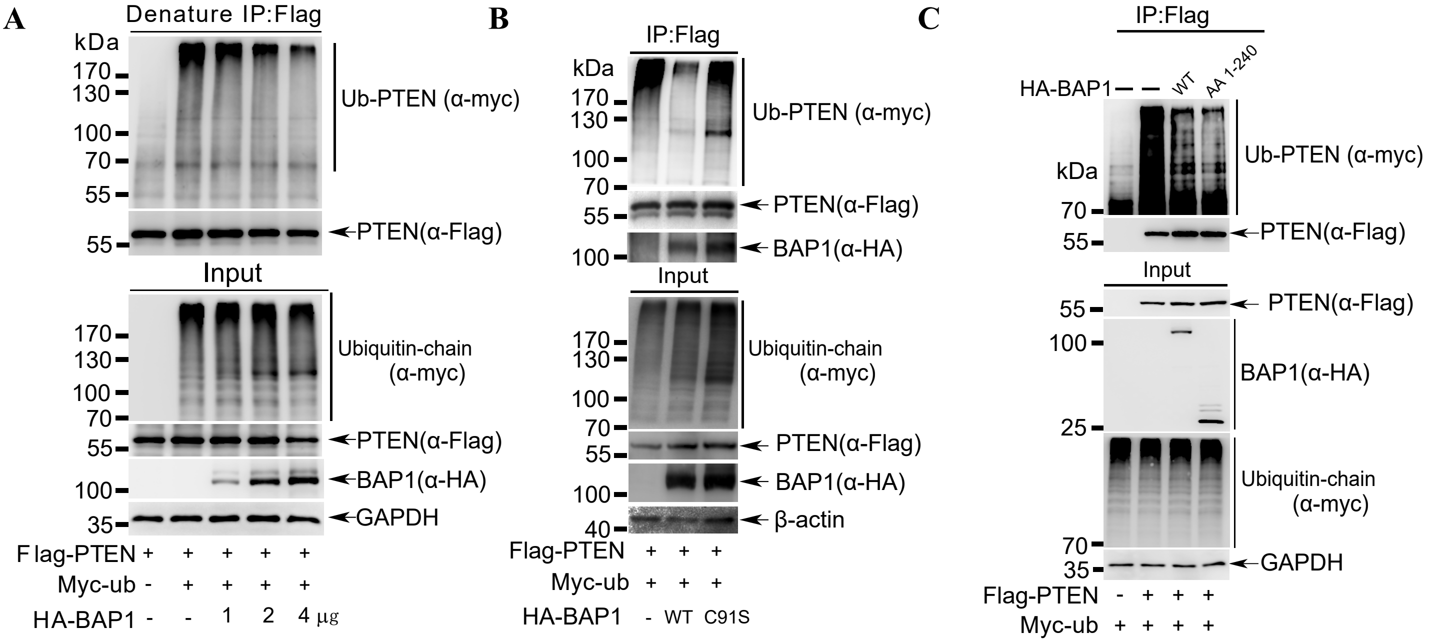

Fig. S5. BAP1 deubiquitinates PTEN. (A) 293T cells were transfected with Flag-PTEN, Myc-Ubiquitin and an increasing amount of HA-BAP1. Lysates were used for immunoprecipitattion with anti-Flag antibody under denaturing condition, and followed by Western blotting analysis with anti-Myc antibody. (B) Lysates from 293T cells transfected with Flag-PTEN, Myc-Ubiquitin and $BAP1^{WT}$ or $BAP1^{C91S}$ were immunoprecipitated with anti-Flag antibody, and followed by Western blotting analysis with anti-Myc antibody. (C) Lysates from 293T cells transfected with Flag-PTEN, Myc-Ubiquitin and full-length $HA\text{-}BAP1^{WT}$ or truncated $HA\text{-}BAP1^{1\text{-}240}$ were immunoprecipitated with anti-Flag antibody, and followed by Western blotting analysis with anti-Myc antibody.
