# Supplementary material for: BAP1 suppresses prostate cancer progression by deubiquitinating and stabilizing PTEN
Source: Mol Oncol. 2020 Nov 20;15(1):279–98. doi: 10.1002/1878-0261.12844 (PMC7782096; doi:10.1002/1878-0261.12844)
Supplement: Supplementary file 6 — Fig. S6. Downregulated PTEN expression promotes activation of Akt pathway and PCa cell colony formation. [file MOL2-15-279-s006.pdf]

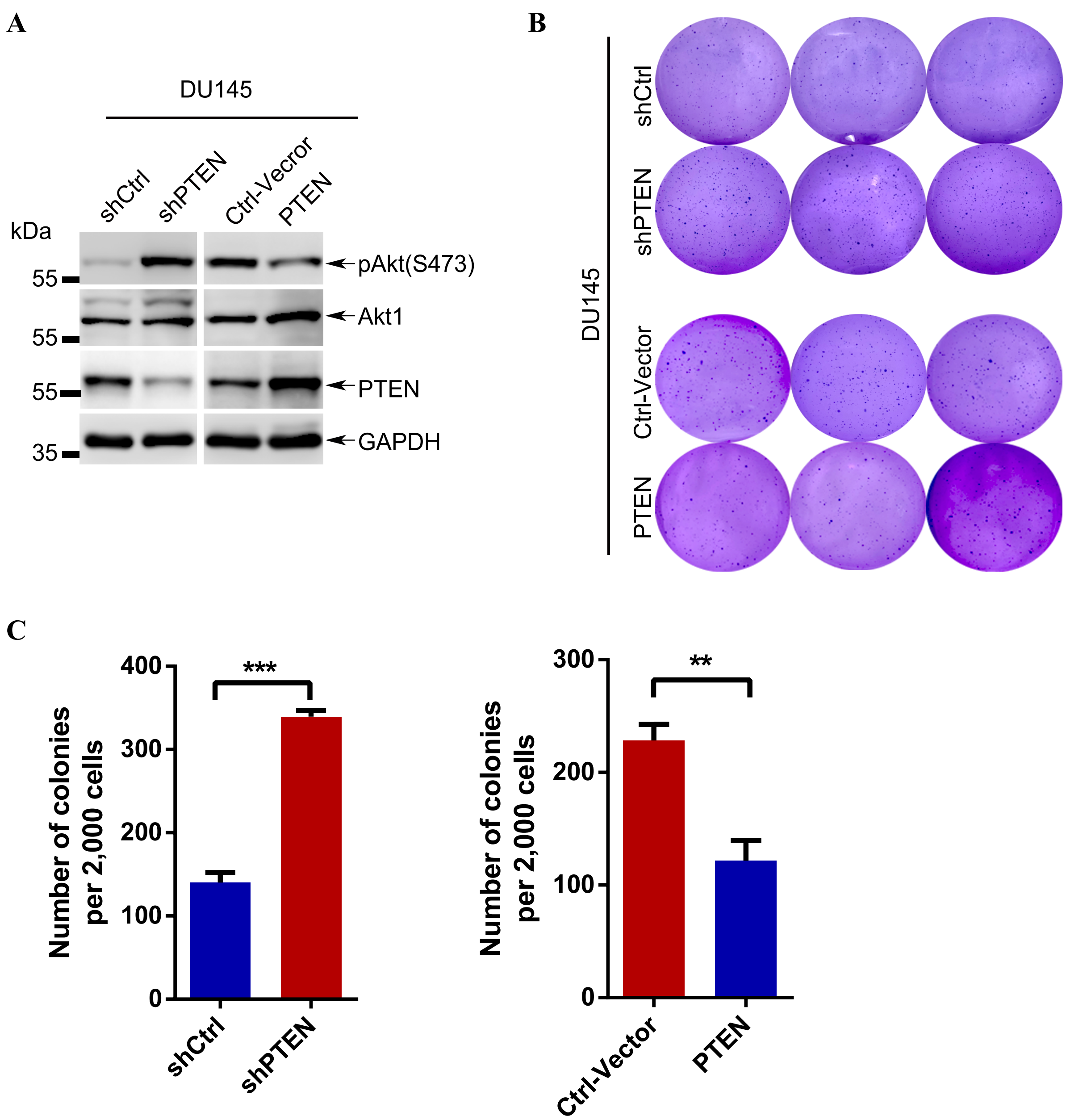

Fig. S6. Downregulated PTEN expression promotes activation of Akt pathway and PCa progression. (A) Western blotting analysis for p-Akt (Ser473) in DU145 stable cells with PTEN knockdown or overexpression. (B-C) Soft agar colony formation assays for DU145 stable cell lines. The representative photographs of colonies were taken (B), and the number of colonies was scored (C). Error bars indicated mean ± SD. Data analysis was conducted by unpaired t-test (*P < 0.05, **P < 0.01, ***P < 0.001).
