# Supplementary material for: BAP1 suppresses prostate cancer progression by deubiquitinating and stabilizing PTEN
Source: Mol Oncol. 2020 Nov 20;15(1):279–98. doi: 10.1002/1878-0261.12844 (PMC7782096; doi:10.1002/1878-0261.12844)
Supplement: Supplementary file 7 — Fig. S7. BAP1 inhibits tumorigenesis through maintenance of PTEN. [file MOL2-15-279-s007.pdf]

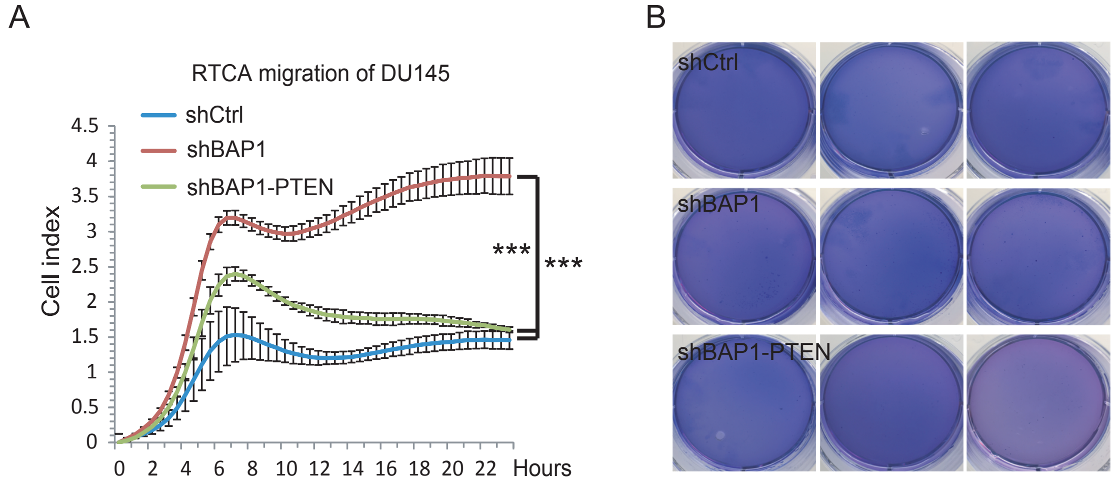

Fig. S7. BAP1 inhibits tumorigenesis through maintenance of PTEN. (A) Stable DU145 cell lines were serum starved for 4-6 hours, then detached with trypsin and resuspended in serum-free medium with concentration of 1×10^5 cells per ml. 100 μl of suspension was seeded into the pre-equilibrated upper chamber of the CIM-plate along with the bottom well containing complete medium for migration. Cell index values were detected every 15 min. Error bars indicate ±SD. These are related to Fig.6C. (B) Stable DU145 cell lines were seeded in 2 ml of medium containing 5% FBS with 0.35% agar at 2000 cells/well. The number of colonies was scored. These are related to Fig. 6E. The DU145 stable cells with BAP1 knockdown were established by using BAP1-shRNA-1#.
