# Supplementary material for: BAP1 suppresses prostate cancer progression by deubiquitinating and stabilizing PTEN
Source: Mol Oncol. 2020 Nov 20;15(1):279–98. doi: 10.1002/1878-0261.12844 (PMC7782096; doi:10.1002/1878-0261.12844)
Supplement: Supplementary file 8 — Fig. S8. BAP1 suppresses PCa progression in an Akt‐dependent manner. [file MOL2-15-279-s008.pdf]

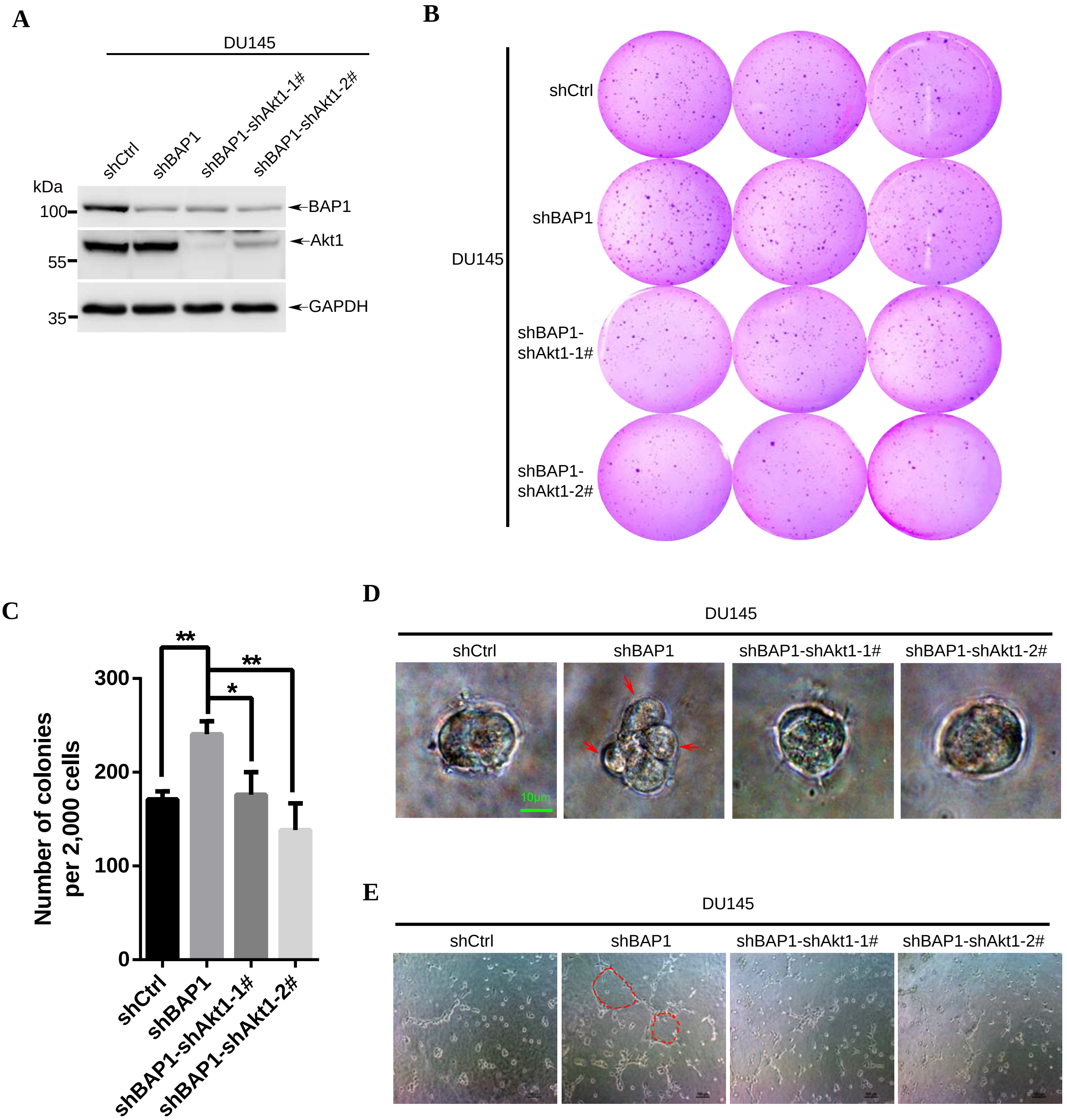

Fig. S8. BAP1 suppresses PCa progression in a Akt1-depedent manner. (A) The efficiency of BAP1 and Akt1 knockdown in DU145 stable cells used in Fig. S6. BAP1 and Akt1 protein levels were determined by using Western blotting analysis. GAPDH was used as internal control. (B-C) Soft agar colony formation assays for DU145 stable cells with BAP1 knockdown or BAP1/Akt1 double knockdown . The representative photographs of colonies were taken (B) and the number of colonies was scored (C). (D) 3D cell culture assays for DU145 stable cell lines. Representative pictures were taken at 4 days. Scale bars: 10 μm. (E) Vasculogenic mimicry assays for DU145 stable cell lines. Representative pictures were taken. Scale bars: 100 μm. BAP1-shRNA-1# was used for the BAP1 knockdown in DU145 cells used in Fig. S8A-E.
