# Supplementary material for: BAP1 suppresses prostate cancer progression by deubiquitinating and stabilizing PTEN
Source: Mol Oncol. 2020 Nov 20;15(1):279–98. doi: 10.1002/1878-0261.12844 (PMC7782096; doi:10.1002/1878-0261.12844)
Supplement: Supplementary file 9 — Table S1. List of DNA or RNA oligonucleotides. Table S2. The list of clinical cancers used for correlation analysis. [file MOL2-15-279-s009.docx]

**Table S1: List of DNA or RNA oligonucleotides**

| Names | Oligonucleotides (5’- 3’) |
| --- | --- |
| BAP1-F | ccggaattcatgaataagggctggctggagctg |
| BAP1-R | ataagaatgcggccgctcactggcgcttggccttgtaggg |
| BAP1^C91S^-F | agtgcaactcatgccttgctgagc |
| BAP1^C91S^-R | agagttgggtatcagctggtggg |
| HA-BAP1(1-240)-F | tgagcggccgctcgagtctagagg |
| HA-BAP1(1-240)-R | cttgatcctgcggtcgggcaccac |
| HA-BAP1(241-596)-F | ccggaattctatgaggccaggctgcatgtgctg |
| HA-BAP1(241-596)-R | ataagaatgcggccgctcagctggacccctggctgccttgg |
| HA-BAP1(597-729)-F | agcccagtggagaaggaggtcgtg |
| HA-BAP1(597-729)-R | gaattccaccacactggatcctgc |
| HA-BAP1^ΔNLS^-F | ccataagtggagaaggaggtcgtggaagccacggacagc |
| HA-BAP1^ΔNLS^-R | ctccacttatgggctgctggacccctggctgccttggat |
| Flag-PTEN(1-350)-F | tgagtcgactctagaggatcccg |
| Flag-PTEN(1-350)-R | tgtttttgtgaagtacagcttcacc |
| Flag-PTEN(1-400)-F | tgagtcgactctagaggatcccg |
| Flag-PTEN(1-400)-R | aatttgtgtatgctgatcttcatcaa |
| Flag-PTEN(188-403)-F | tatagaccagtggcactgttgtttc |
| Flag-PTEN(188-403)-R | cttgtcatcgtcgtccttgtagtccat |
| shBAP1-1#-F | ccggccctcagtattaccatgtctctcgagagacatggtaatactgagggtttttg |
| shBAP1-1#-R | aattcaaaaaccctcagtattaccatgtctctcgagagacatggtaatactgaggg |
| shBAP1-2#-F | ccggatcatgccacggtcccaactactcgagtagttgggaccgtggcatgattttttg |
| shBAP1-2#-R | aattcaaaaaatcatgccacggtcccaactactcgagtagttgggaccgtggcatgat |
| shAKT1-1#-F | ccggtcgcgtgaccatgaacgagtttctcgagaaactcgttcatggtcacgcgtttttg |
| shAKT1-1#-R | aattcaaaaacgcgtgaccatgaacgagtttctcgagaaactcgttcatggtcacgcgaa |
| shAKT1-2#-F | ccggtgatcctcaagaaggaagtcatctcgagatgacttccttcttgaggatctttttg |
| shAKT1-2#-R | aattcaaaaagatcctcaagaaggaagtcatctcgagatgacttccttcttgaggatca |
| shCtrl-F | ccggttctccgaacgtgtcacgtctcgagacgtgacacgttcggagaatttttg |
| shCtrl-R | aattcaaaaattctccgaacgtgtcacgtctcgagacgtgacacgttcggagaa |
| siBAP1.214 | cgaccuucagagcaaaugu |
| siBAP1.2132 | gaguucaucugcaccuuua |
| QPCR-PTEN-F | gatgaggcattatcctgtacaca |
| QPCR-PTEN-R | ctcttcagatactcttgtgctgt |
| QPCR-GAPDH-F | atgaggtccaccaccctgtt |
| QPCR-GAPDH-R | ctcaagggcatcctgggcta |
|  |  |
|  |  |

**Table S2: The list of clinical cancers used for correlation analysis**

| Data Sets | Samples(n) | Correlation | P-value |
| --- | --- | --- | --- |
| TCGA Esophageal carcinoma(ESCA) | 126 | 0.544 | 0 |
| TCGA Kidney Chromophobe(KICH) | 63 | 0.5084 | 0.000027366 |
| TCGA Bladder Urothelial Carcinoma(BLCA) | 344 | 0.50674 | 7.71E-24 |
| TCGA Stomach adenocarcinoma(STAD) | 392 | 0.49095 | 3.59E-25 |
| TCGA Thymoma(THYM) | 90 | 0.48929 | 1.3821E-06 |
| TCGA Sarcoma(SARC) | 221 | 0.43877 | 8.17E-12 |
| TCGA Pancreatic adenocacinoma(PAAD) | 105 | 0.39087 | 0.000043175 |
| TCGA Prostate adenocarcinoma(PRAD) | 351 | 0.38405 | 8.82E-14 |
| TCGA Head and Neck squamous cell carcinoma  (HNSC) | 346 | 0.32112 | 9.70E-10 |
| TCGA Cervical squamous cell carcinoma and  endocervical adenocarcinoma(CESC) | 171 | 0.2988 | 0.000077494 |
| TCGA Liver hepatocellular carcinoma(LIHC) | 184 | 0.28857 | 0.000071128 |
| TCGA Kidney renal papillary cell carcinoma(KIRP) | 208 | 0.2724 | 0.000073154 |
| TCGA Breast invasion carcinoma(BRCA) | 901 | 0.22697 | 1.13E-11 |
| TCGA Thyroid carcinoma(THCA) | 374 | 0.20649 | 0.000060054 |
| TCGA Skin Cutaneous Melanoma(SKCM) | 354 | 0.19266 | 0.0019097 |
| TCGA Lung squamous cell carcinoma(LUSO) | 325 | 0.17017 | 0.0021071 |
| TCGA Lung adenocarcinoma(LUAD) | 362 | 0.13838 | 0.0083773 |
| TCGA Kidney renal clear cell carcinoma(KIRC) | 445 | 0.11965 | 0.011535 |
| TCGA Brain Lower Grade Glioma(LGG) | 427 | -0.18319 | 0.00014083 |
| TCGA Testicular Germ Cell Tumors(TGCT) | 118 | -0.29772 | 0.0011098 |
| TCGA Cholangiocarcinoma(CHOL) | 30 | 0.30145 | 0.10564 |
| TCGA Uveal Melanoma(UVM) | 12 | 0.26573 | 0.40398 |
| TCGA Lymphoid Neoplasm Diffuse Large B-cell  Lymphoma(DLBC) | 33 | 0.22761 | 0.20195 |
| TCGA Uterine Carcinsarcoma(UCS) | 48 | 0.17531 | 0.23254 |
| TCGA Mesothelioma(MESO) | 61 | 0.074644 | 0.56751 |
| TCGA Glioblastoma multiforme(GBM) | 205 | 0.067141 | 0.33881 |
| TCGA Adrenocortical carcinoma(ACC) | 46 | 0.022633 | 0.88115 |
| TCGA Uterine Corpus Endometrial Carcinoma  (UCEC) | 404 | 0.013672 | 0.78411 |
| TCGA Pheochromocytoma and Paraganglioma  (PCPG) | 81 | -0.020472 | 0.85763 |
| TCGA Ovarian serous cystadencarcinoma(OV) | 411 | -0.053006 | 0.28355 |
| TCGA Colon adenocarcinoma(COAD) | 358 | -0.091778 | 0.08377 |
| TCGA Rectum adenocarcinoma(READ) | 130 | -0.16392 | 0.062453 |
